# Examination under anesthesia imaging changes surgeons’ classification and treatment decisions of anterior posterior compression pelvic ring injuries

**DOI:** 10.1007/s00590-026-04744-8

**Published:** 2026-05-30

**Authors:** Camryn C. Therrien, Kaj ten Duis, Alessandro Aprato, Hester Banierink, Johannes D. Bastian, Andreas Höch, Umberto M. R. Mezzadri, Jakob van Oldenrijk, Philipp Pieroh, Henry C. Sagi, Jean-Paul P. M. de Vries, Inge H. F. Reininga, Frank F. A. IJpma, Society of European Pelvic and Acetabulum Surgeons, Kamarul Arifin Abdul Razak, Francesco Addevico, Yaqoub Alotheri, José Vicente Andrés-Peiró, Charlotte Arand, Ahmad Arieff Atan, Mark C. P. M. van Baal, Sebastiano Barreca, Johannes Dominik Bastian, Peter Bates, Taco Bijlsma, Eftychios Bolierakis, Juan Boluda-Mengod, Mehdi Boudissa, Federico Bove, Jeroen Bransen, Pauline Buteau, Alessandro Casiraghi, Stefano Cattaneo, Federico Chiodini, Ferdinand Christian Wagner, Carlo Colonna, Andrej Cretnik, Pietro De Biase, Kaj ten Duis, Michael J. R. Edwards, Daphne van Embden, Benjamin Erdle, Jochen Franke, Abramo Fratus, Paul Kong Fu-Xiang, Axel Gamulin, Axel Gänsslen, Leo M. G. Geeraedts, Antonio Gilli, Miguel Ángel Giráldez-Sánchez, Tjebbe Hagenaars, Maximilian Hartel, Svenhjalmar van Helden, Steven C. Herath, Erik Hermans, Michiel Herteleer, Nico Hinz, Andreas Höch, Mike Hogervorst, Klemens Horst, Aitor Ibarzábal-Gil, Frank F. A. IJpma, Eduardo Jose Burgos, Muhammad Ade Junaidi, Nikolaos Kanakaris, Christian Kleber, Wolfgang Lehmann, Björn-Christian Link, Emmanouil Liodakis, Dario Lo Re, Jean G. Louka, Lapo De Luca, Jan Erik Madsen, Sven Märdian, Arduini Mario, Giovanni Materazzi, Alberto Maurizio, Jan Peter van Meirhaeghe, Sven A. Meylaerts, Umberto M. R. Mezzadri, Ben Molenaers, Antonio Moretti, Jevgenijs Movcans, Josep M. Muñoz-Vives, Muhammad Yasir Bin Ahmad Muslim, Robert J. Nijveldt, Dmitry Notov, Jakob van Oldenrijk, Beatriz Olías-López, Georg Osterhoff, Salvatore Pantè, Raffaele Pascarella, Roman Pfeifer, Philipp Pieroh, Raúl G. Plomp, Tim Pohlemann, Nicolas Poinot, Kees Jan Ponsen, Guy Putzeys, Victor A. De Ridder, Pol M. Rommens, Pieter-Jan De Roo, Marco Santavicca, Emmanuele Santolini, Inger B. Schipper, Uwe Schweigkofler, Michal Stibor, Sander F. L. van Stigt, Vincent Stirler, Charles Tatiana, Jordi Teixidor-Serra, Michel Paul Johan Teuben, Andreas Thannheimer, Frandin Thomas, Andres Torrejón, Viju Daniel Varghese, Jan Verbruggen, Luigi Branca Vergano, Fabricio Videla, Bas van Wageningen, Daniel Wagner, Peer van der Zwaal

**Affiliations:** 1 Department of Trauma Surgery, University of Groningen, University Medical Center Groningen, Groningen, Netherlands; 2 Department of Orthopaedics, University of Turin, Turin, Italy; 3 Department of Orthopaedic Surgery and Traumatology, University Hospital of Bern, Bern, Switzerland; 4 Department of Orthopaedics, Trauma and Plastic Surgery, University Hospital Leipzig, Leipzig, Germany; 5 Department of Orthopaedic and Trauma Surgery, ASST GOM Niguarda, Milan, Italy; 6 Department of Orthopaedics and Sports Medicine, Erasmus MC, Rotterdam, Netherlands; 7 Department of Orthopedic Surgery and Sports Medicine, University of Cincinnati, Cincinnati, United States; 8 Department of Surgery, University of Groningen, University Medical Center Groningen, Groningen, Netherlands

**Keywords:** Anterior-posterior compression injury, Examination under anesthesia, Pelvic stability, Pelvic fixation

## Abstract

**Purpose:**

To report on the effect that Pelvic Examination Under Anesthetic (EUA) has on modifying both the classification and treatment plan for Tile-B, Anterior Posterior Compression (APC) injuries.

**Methods:**

Four hundred international pelvic surgeons were invited to participate in a two-part online survey. Each of the two surveys included the same ten cases selected to represent a spectrum of APC injuries, with the first survey containing only pre-operative static radiographic and CT imaging, and the second survey including the additional EUA images. Surgeons were asked to answer questions regarding classification and treatment.

**Results:**

One hundred twenty-three pelvic surgeons participated (response rate = 31%). Respondents were primarily trauma surgeons (76%) from level 1 trauma centers (82%), with an average of ten (IQR 5–15) years of experience in treating pelvic injuries. The addition of EUA imaging resulted in 46% of the respondents in a change in classification, primarily shifting from APC1 (26% with static imaging to 17% with EUA imaging) to APC2 (57% to 67%). Treatment decisions changed in 36% of responses: non-operative management decreased from 15% to 7%, and anterior-only fixation increased from 22% to 35%.

**Conclusion:**

The addition of EUA images in external rotation pelvic ring injuries altered almost half of the surgeons’ perceptions of the classification (46%) and–more importantly–over one-third (36%) of the surgeons’ treatment plans. The addition of the EUA imaging had its most significant impact on revising injuries initially classified as APC1, indicating an underestimation of injury severity in this group. However, these changes varied widely across the representative injuries, with EUA serving as a confirmatory tool in some cases, while revealing occult instability in others.

**Supplementary Information:**

The online version contains supplementary material available at 10.1007/s00590-026-04744-8.

## Introduction

Anterior-posterior compression (APC) pelvic ring injuries occur due to external rotational forces on the pelvis, frequently resulting in disruption of the pubic symphysis, the sacrospinous and sacrotuberous ligaments, and the anterior and posterior sacroiliac (SI) ligaments [1]. APC injuries are classified as APC1, APC2, or APC3, depending on the extent of sacroiliac (SI) ligament involvement [2].

Classification of pelvic ring injuries is a recognized challenge for even experienced pelvic surgeons [3–6]. This is due, in part, to the notion that the extent to which the various structures (bones, musculature, and supporting ligaments) in the pelvic ring are injured occurs along a dynamic spectrum. Standard radiographs and computed tomographic (CT) scans used for initial assessment and treatment planning provide only a static evaluation, which does not always represent the maximal degree of displacement or instability. Moreover, the use of pelvic binders or circumferential sheeting during resuscitation has been shown to obscure the degree of displacement [7, 8], which can result in an underestimation of the level of pelvic ring instability, potentially resulting in improper treatment decisions [3, 4, 9–11]. Specifically, hardware complications, such as screw loosening or anterior plate failure, are relatively common following fixation of APC injuries and may reflect underestimation of injury severity on static imaging [12, 13].

A protocol for dynamic stress examination under anesthesia (EUA) with fluoroscopy was introduced by Sagi et al. to evaluate pelvic ring stability [3], revealing occult instability in APC injuries that would otherwise be missed using static radiography alone [3, 4]. Accurate dynamic assessment of both anterior and posterior pelvic ring instability in APC injuries is essential for determining the appropriate reduction and fixation strategy. Underestimation of the full extent of instability increases the risk of subsequent displacement, malunion and non-union if inadequate fixation strategies are employed. For some surgeons, EUA can help to guide the decision between nonoperative management or various permutations of anterior and/or posterior fixation.

While displacement on EUA has been proposed to guide both classification and treatment (Appendix 1) [3], this technique remains controversial because the method has not yet been validated with respect to standardized parameters and functional outcomes [14]. Furthermore, although EUA has been shown to improve agreement between pelvic surgeons regarding instability and the need for mechanical stabilization in patients with Lateral Compression injuries (LC) [15], this effect has not been specifically evaluated in APC injuries. Therefore, it is important to investigate how EUA influences surgeons’ injury classification and operative decision-making in patients with APC pelvic ring injuries.

Accordingly, an international survey of pelvic surgeons was conducted to answer the following research questions:

1. Does the addition of EUA images change the surgeon’s classifications of APC pelvic ring injuries?
2. Does the addition of EUA images change the surgeon’s treatment decisions in patients with APC pelvic ring injuries?

## Methods

An international study was conducted to evaluate surgeons’ classification and treatment decisions for APC pelvic ring injuries using two separate surveys. The first survey contained only static radiographic (Antero-Posterior (AP), inlet and outlet projections) and CT imaging of ten patients with external rotation pelvic ring injuries. One month later, a second survey containing the same ten cases was re-presented with the addition of dynamic EUA images. Participating surgeons were asked to provide both fracture classification and a treatment recommendation for each case. All cases were selected by experienced pelvic surgeons to reflect a full spectrum of Young and Burgess APC injury severity (stable, borderline, and clearly unstable patterns).

The local Medical Ethical Review Board reviewed the methods employed and waived further need for approval (METc 2017/543).

### Case description

The case selection criteria were adult patients who had an APC injury [1] based on assessment of the preoperative static radiographic and CT imaging, all of whom underwent EUA with fluoroscopy. To have a more accurate representation of true clinical practice, five of the ten cases were initially managed with pelvic binders during pre-operative CT imaging (binder closed and tightened), and five were managed without a binder [16]. All ten cases had static AP radiographs taken without pelvic binders in place. Static and dynamic images for all ten cases are contained in Appendix 2.

### Surveys

Four hundred pelvic surgeons were invited to participate in this online survey via email in March of 2025. The REDCap (Research Electronic Data Capture) data collection platform was used for the initial invitation and the distribution of all surveys throughout this study. REDCap is a secure, web-based software platform designed to support data capture for research studies [17, 18].

Apart from the ten cases, the first survey included general questions regarding the surgeon’s individual experience regarding pelvic surgery and clinical practice (Appendix 3). Thereafter, each case was presented, including a static AP radiograph and an axial CT scan of each patient, followed by a series of questions about classification and treatment (Appendix 4). The same questions were asked for all cases.

The second survey included the same ten cases presented in a different order than the first survey. The order of case presentation was the same for all surgeons. This survey included the same static images as in the first survey, with the addition of the following four AP EUA images: internal rotation stress (lateral compression), external rotation stress (frog-leg position), push right leg/ pull left leg, and push left leg/ pull right leg [3]. Rather than including the full series of 15 EUA images [3], we limited the number of dynamic views to four representative stress maneuvers to enhance clarity and reproducibility of interpretation for respondents while maintaining the clinically relevant information needed to assess pelvic ring instability. Each EUA image included a radiopaque ruler, allowing surgeons to take measurements if desired. The same questions were asked for all cases (Appendix 4).

### Statistical analysis

For Gaussian distributed data, means and standard deviations (SD) were presented, and for non-Gaussian distributed data, medians and interquartile ranges (IQR) were used. Frequency was presented as n (% of all patients). The statistical analysis was performed using IBM SPSS Statistics (Version 28).

The following treatment groups were made: non-operative, anterior-only fixation, and both anterior and posterior fixation, based on the specific treatment decisions made by the surgeons. If the surgeon’s response did not fit within one of these three groups, it was recorded as ‘other’.

Whether the surgeon’s classification or treatment decision differed on the second survey compared to the first survey was recorded as a changed classification or treatment decision. The number of changed classifications and treatment decisions is presented for each case and in total across the ten cases. Furthermore, all the classifications and treatment decisions from both surveys are presented per case.

Sub-analyses were performed to determine if there were differences in the number of changed classifications and treatment decisions between surgeons who perform EUA in their standard practice and those who do not, and also between surgeons who treat 50 or more pelvic ring injuries per year and those who treat fewer than 50 per year. The Chi-Square Test was used for these analyses, with a significance set at a p-value of < 0.05.

## Results

### Surgeons’ experience and practice

Of the four hundred surgeons solicited, one hundred twenty-three surgeons (31%) agreed to participate in this survey. Information regarding their experience and standard practice is presented in Table 1.

**Table 1:** Surgeon information

Surgeon information (*N* = 123)	
Specialty	
Trauma surgery, n (%)	94 (76)
Orthopedic surgery, n (%)	23 (19)
Other*, n (%)	6 (5)
Level of trauma center	
Level 1, n (%)	101 (82)
Level 2, n (%)	15 (12)
Level 3, n (%)	7 (6)
Years practicing specialty, median (IQR)	14 (6–30)
Years treating pelvic ring injuries, median (IQR)	10 (5–15)
Pelvic injuries treated per year, median (IQR)	50 (20–70)
Uses EUA in their standard practice, n (%)	61 (50)
Country of practice	
Germany	36 (29)
Italy	25 (20)
Netherlands	18 (15)
Belgium	11 (9)
Spain	7 (6)
Malaysia	4 (3)
United Kingdom	4 (3)
Switzerland	4 (3)
France	2 (2)
Argentina	2 (2)
United States	2 (2)
Saudi Arabia	1 (1)
Latvia	1 (1)
Andorra	1 (1)
Norway	1 (1)
Indonesia	1 (1)
India	1 (1)
Cameroon	1 (1)
Slovenia	1 (1)
Austria	1 (1)
Australia	1 (1)

*Combination of orthopedic and trauma (*n* = 5), military and trauma surgery (*n* = 1)

### Changes in classification

**Fig. 1:**
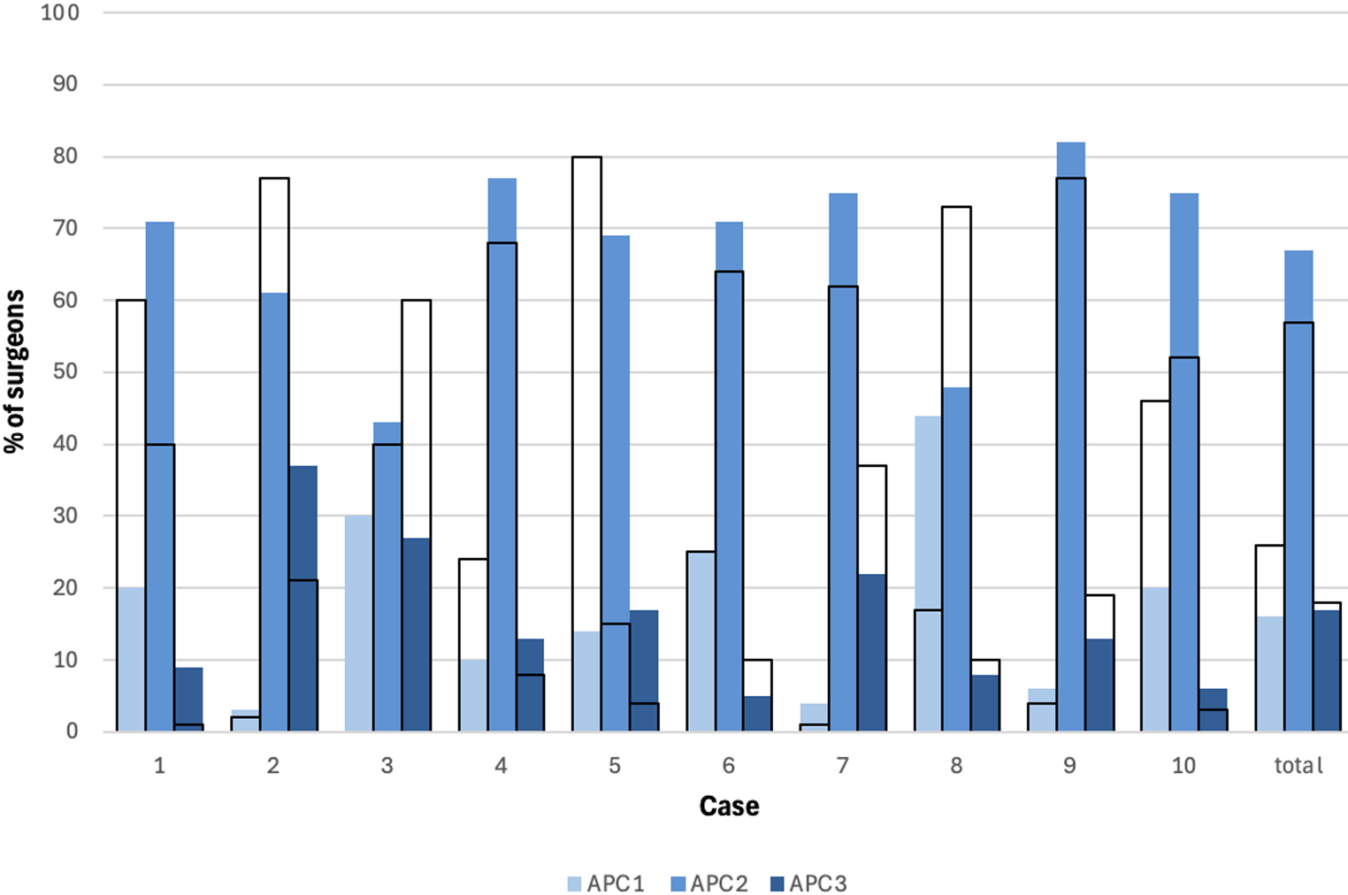
Classifications of each case based on static radiographs and CT-scans (first survey; transparent bars outlined in black) compared to classifications based on EUA (second survey; blue bars)

**Fig. 2:**
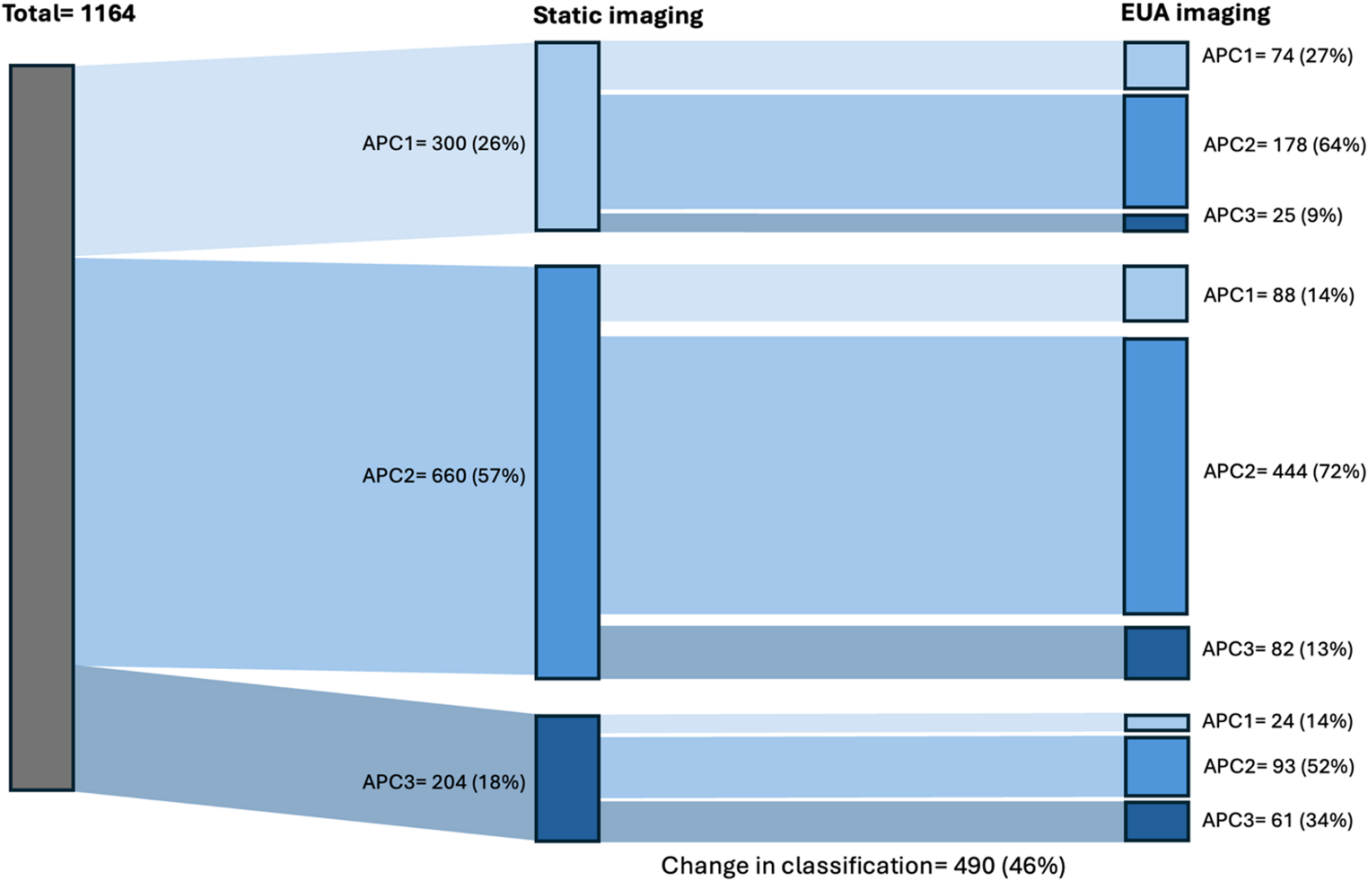
Changes in classification after the addition of EUA imaging

The classifications (APC1, APC2, or APC3) selected by the surgeons based on the static imaging and the EUA imaging are presented in Table 2; Fig. 1, and the rates of change of classification between surveys are presented in Table 2. Various shifts of classification can be observed in Figs. 1 and 2. With all cases combined, an overall shift from APC1 to APC2 can be observed between surveys, going from 26% APC1 and 57% APC2 based on static imaging to 17% and 67% respectively, based on EUA imaging. The rates at which classifications differed between static and dynamic images varied considerably across cases, ranging from 28% (*n* = 31) to 76% (*n* = 82).

**Table 2:** Classifications for each case based on static radiographs and CT-scans (survey 1) and EUA imaging (survey 2)

Case*	Total	1	2	3	4	5	6	7	8	9	10
Survey 1: Static images	(n = 1164)	(n = 119)	(n = 119)	(n = 118)	(n = 118)	(n = 117)	(n = 115)	(n = 115)	(n = 115)	(n = 114)	(n = 114)
APC1 n (%)	300 (26)	71 (60)	2 (2)	0	28 (24)	94 (80)	29 (25)	1 (1)	19 (17)	4 (4)	52 (46)
APC2 n (%)	660 (57)	47 (40)	92 (77)	47 (40)	80 (68)	18 (15)	74 (64)	71 (62)	84 (73)	88 (77)	59 (52)
APC3 n (%)	204 (18)	1 (1)	25 (21)	71 (60)	10 (8)	5 (4)	12 (10)	43 (37)	12 (10)	22 (19)	3 (3)
Survey 2: AP EUA images	(n = 1070)	(n = 106)	(n = 109)	(n = 106)	(n = 107)	(n = 108)	(n = 106)	(n = 106)	(n = 108)	(n = 108)	(n = 106)
APC1 n (%)	186 (17)	21 (20)	3 (3)	32 (30)	11 (10)	15 (14)	26 (25)	4 (4)	47 (44)	6 (6)	21 (20)
APC2 n (%)	716 (67)	75 (71)	66 (61)	45 (43)	82 (77)	75 (69)	75 (71)	79 (75)	52 (48)	88 (82)	79 (75)
APC3 n (%)	168 (17)	10 (9)	40 (37)	29 (27)	14 (13)	18 (17)	5 (5)	23 (22)	9 (8)	14 (13)	6 (6)
Change of classification, n (%)	490 (46)	60 (57)	44 (40)	63 (59)	41 (38)	82 (76)	31 (29)	39 (37)	49 (42)	31 (28)	50 (47)

*Case numbers that had a pelvic binder on during CT scans are underlined

### Changes in treatment decisions

**Fig. 3:**
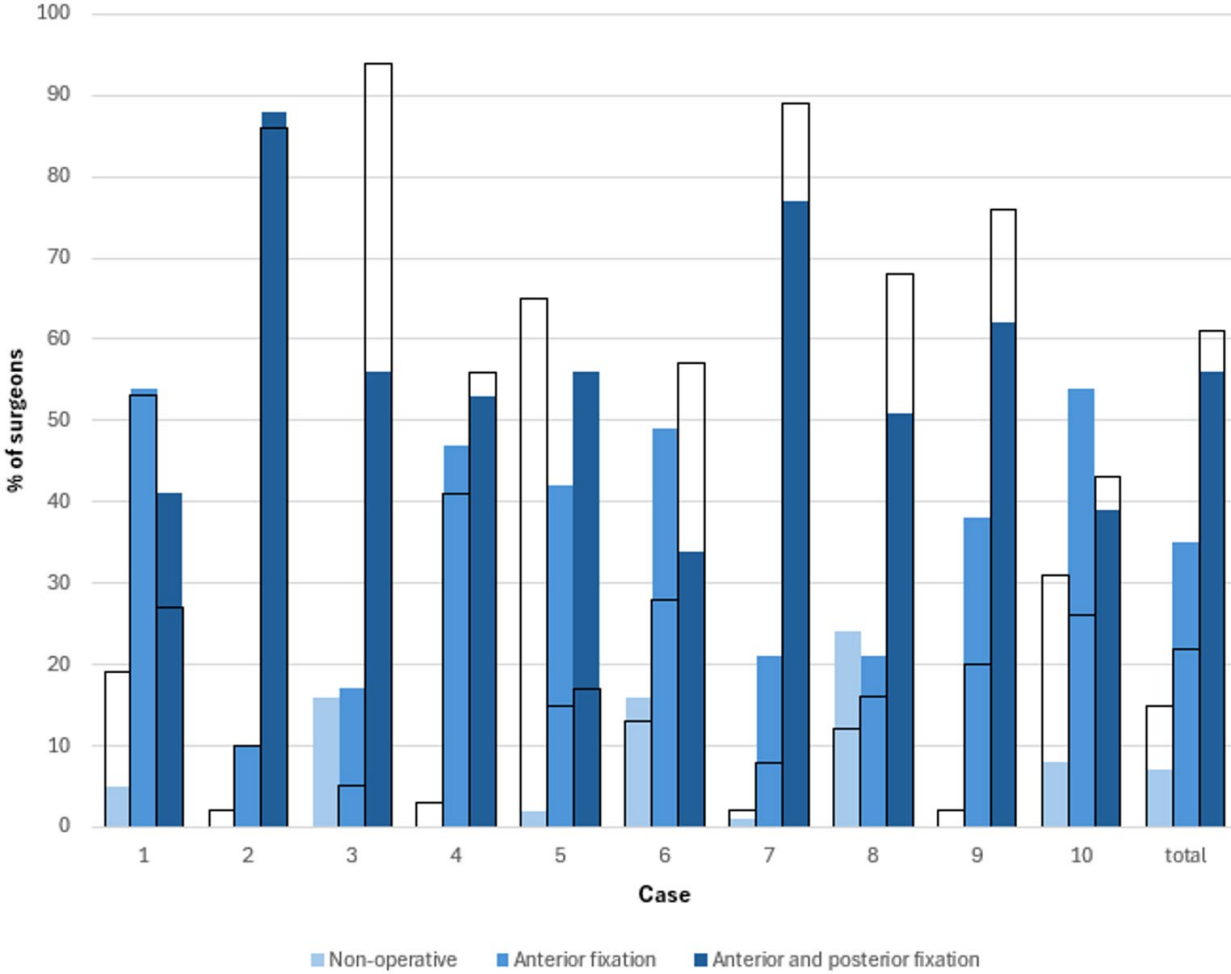
Treatment decisions for each case based on static radiographs and CT-scans (first survey; transparent bars outlined in black) compared to classifications based on EUA (second survey; blue bars)

**Fig. 4:**
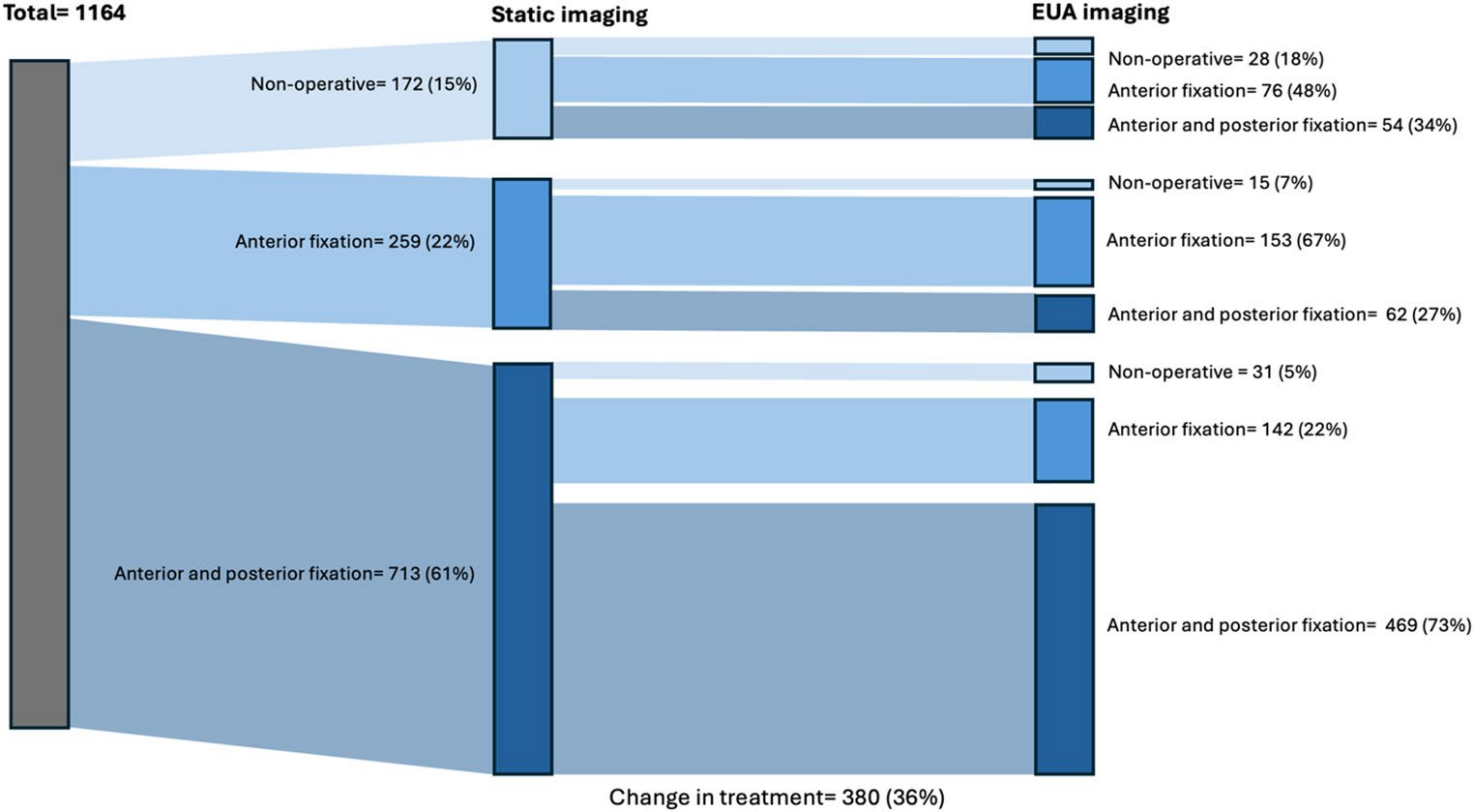
Changes in treatment decision after the addition of EUA imaging. The treatment choice “other” is not represented in this figure; refer to Table 3

The treatment decisions (non-operative, anterior only, or combined anterior and posterior fixation) selected by the surgeons based on the static imaging and the EUA imaging are presented in Table 3; Fig. 3, and the rates of change in classification between surveys are presented in Table 3. With all cases combined, the treatment decisions shifted away from a non-operative approach, towards an anterior-only fixation approach (Fig. 4). The non-operative treatment approach decreased from 15% to 7%, the anterior-only approach increased from 22% to 35% and the combined anterior and posterior fixation decreased from 61% to 56%. The rates at which treatment decisions differed between static and dynamic images varied considerably across cases, ranging from 13% (*n* = 14) to 78% (84).

**Table 3:** Treatment decisions for each case based on static radiographs and CT-scans (survey 1) and EUA imaging (survey 2)

Case	Total	1	2	3	4	5	6	7	8	9	10
Survey 1: Static images	(n = 1164)	(n* = 119)	(n = 119)	(n = 118)	(n = 118)	(n = 117)	(n = 115)	(n = 115)	(n = 115)	(n = 114)	(n = 114)
Non-operative, n (%)	172 (15)	23 (19)	2 (2)	0 (0)	3 (3)	76 (65)	15 (13)	2 (2)	14 (12)	2 (2)	35 (31)
Anterior fixation only, n (%)	259 (22)	63 (53)	12 (10)	6 (5)	48 (41)	18 (15)	32 (28)	9 (8)	18 (16)	23 (20)	30 (26)
Anterior and posterior fixation, n (%)	713 (61)	32 (27)	102 (86)	111 (94)	66 (56)	20 (17)	66 (57)	102 (89)	78 (68)	87 (76)	49 (43)
Other, n (%)	20 (2)	1 (1)	3 (3)	1 (1)	1 (1)	3 (3)	2 (2)	2 (2)	5 (4)	2 (2)	0
Survey 2: AP EUA images	(n = 1070)	(n = 106)	(n = 109)	(n = 106)	(n = 107)	(n = 108)	(n = 106)	(n = 106)	(n = 108)	(n = 108)	(n = 106)
Non-operative, n (%)	76 (7)	5 (5)	0 (0)	17 (16)	0	2 (2)	17 (16)	1 (1)	26 (24)	0	8 (8)
Anterior fixation only, n (%)	376 (35)	57 (54)	11 (10)	18 (17)	50 (47)	45 (42)	52 (49)	22 (21)	23 (21)	41 (38)	57 (54)
Anterior and posterior fixation, n (%)	596 (56)	43 (41)	96 (88)	59 (56)	57 (53)	60 (56)	36 (34)	82 (77)	55 (51)	67 (62)	41 (39)
Other, n (%)	22 (2)	1 (1)	2 (2)	12 (11)	0	1 (1)	1 (1)	1 (1)	4 (4)	0	0
Change of treatment group, n (%)	380 (36)	45 (42)	14 (13)	34 (32)	34 (32)	84 (78)	38 (36)	22 (21)	31 (31)	32 (39)	46 (43)

*Case numbers that had a pelvic binder on during CT scans are underlined

### Sub-analysis

The sub-analysis of the differences in rates of change of classification and treatment decisions between surgeons who perform EUA (*n* = 61, 50%) and surgeons who do not perform EUA (*n* = 62, 50%) in their standard practice is presented in Appendix 5. In case 4, surgeons who do not use EUA had a higher rate of change compared to EUA users, with 26 (48%) vs. 15 (28%) (*p* = 0.019), and in case 9, surgeons who use EUA had a higher rate of change compared to non-EUA users, with 20 (38%) vs. 11 (21%) (*p* = 0.043) (see imaging for cases in Appendix 2). For all other cases, no significant differences in classification or treatment decision changes were observed.

In the sub analysis of surgeons who treat more than 50 pelvic ring injuries per year (*n* = 67, 55%) vs. those who treat less than 50 per year (*n* = 56, 46%) showed that surgeons who treat fewer injuries per year had a higher rate of change of classification in case 4 with 14 (25%) vs. 27 (54%) (*p* = 0.002) and a higher rate of change of treatment in case 6, with 15 (26%) vs. 23 (50%) (*p* = 0.012) (Appendix 6).

Furthermore, the average rate of change in classification was 45% for cases with pelvic binders compared with 46% for cases without binders. For treatment decisions, the average rate of change was 31% in patients with pelvic binders and 43% in patients without binders. These findings indicate that the observed changes were not confounded by the use of a pelvic binder.

### Case examples

**Fig. 5:**
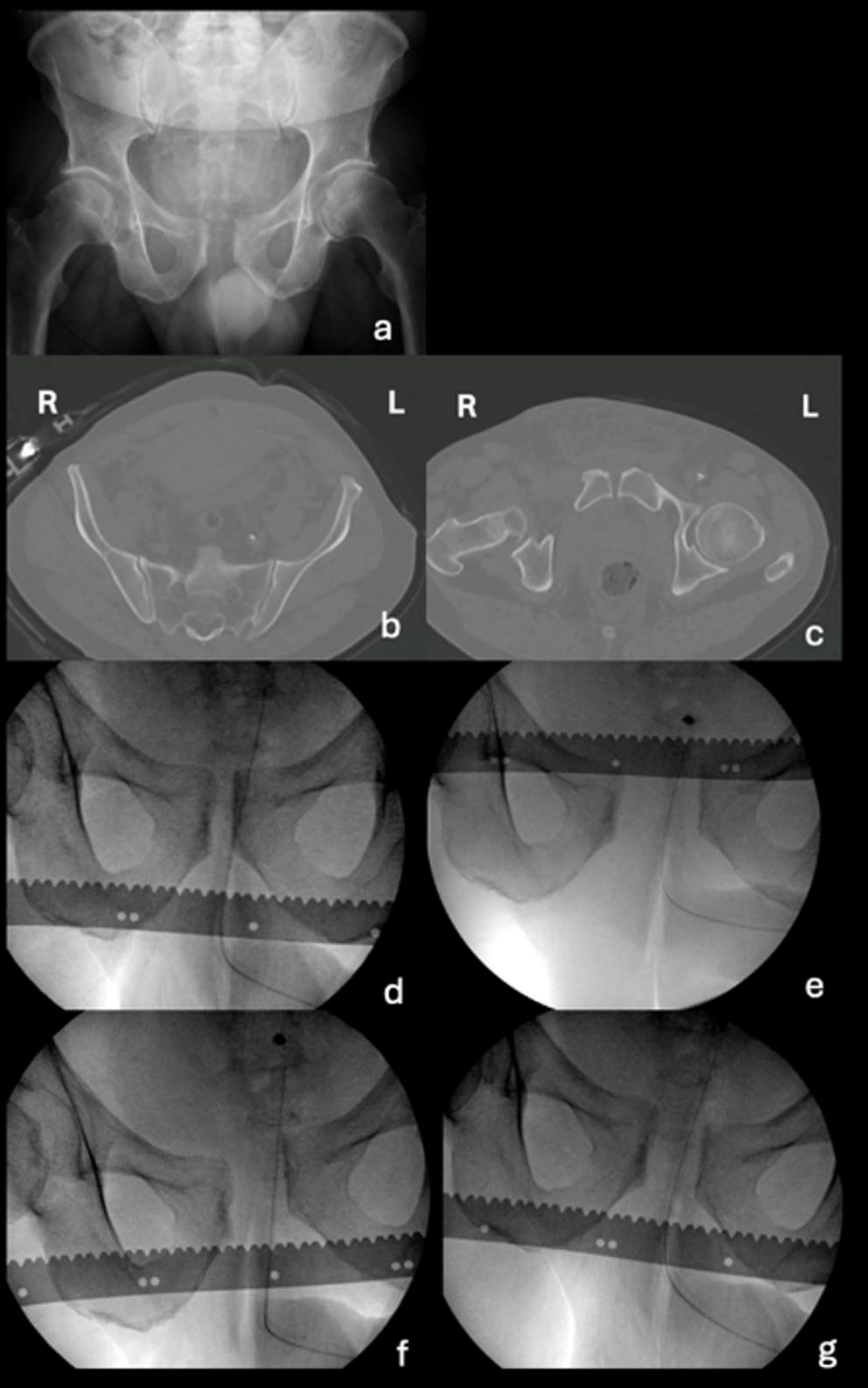
Case 1, **a** AP static radiograph, (**b**) CT scan showing the sacroiliac joint, (**c**) CT scan showing pubic symphysis, (**d**) AP EUA image in endorotation stress (lateral compression), (**e**) AP EUA image in exorotation stress (frog position), (**f**) AP EUA image in push right leg/ pull left leg stress (rotational instability), (**g**) AP EUA image in push left leg/ pull right leg stress (rotational instability)

**Fig. 6:**
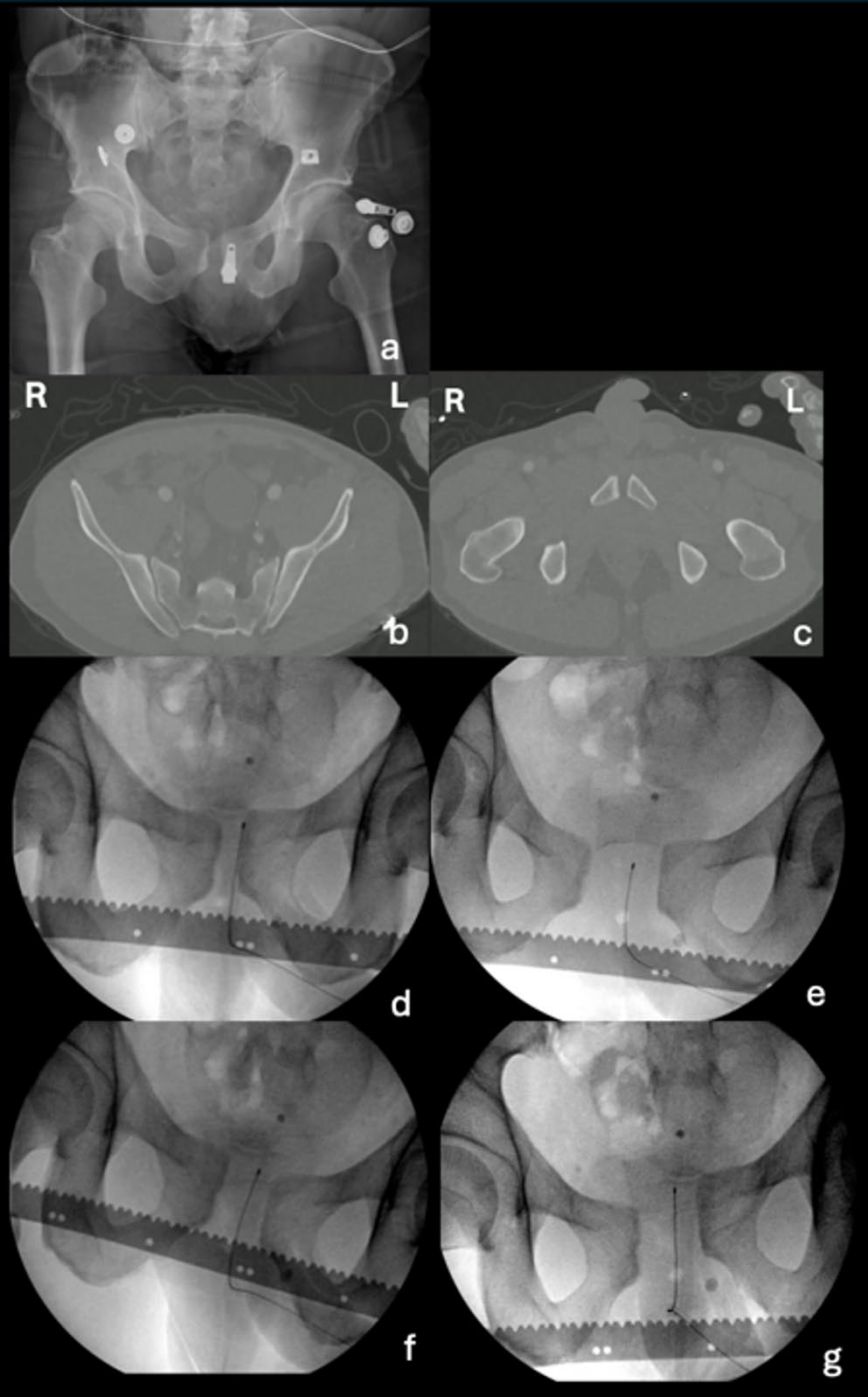
Case 5, **a** AP static radiograph (artifacts on image are pieces of metal from clothing), **b** CT scan showing the sacroiliac joint, **c** CT scan showing pubic symphysis, **d** AP EUA image in endorotation stress (lateral compression), **e** AP EUA image in exorotation stress (frog position), **f** AP EUA image in push right leg/ pull left leg stress (rotational instability), **g** AP EUA image in push left leg/ pull right leg stress (rotational instability)

**Fig. 7:**
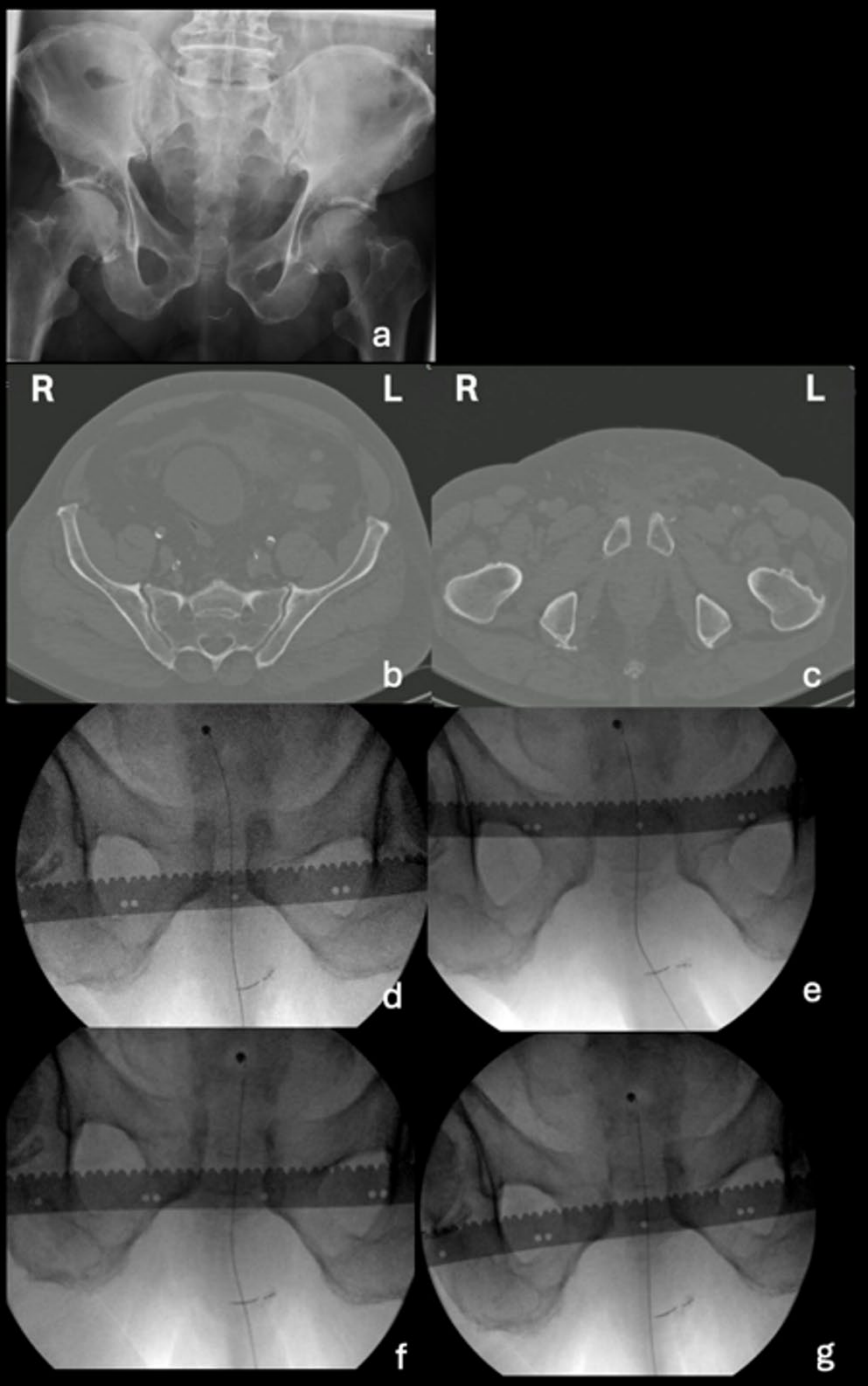
Case 10, **a** AP static radiograph, **b** CT scan showing the sacroiliac joint, **c** CT scan showing pubic symphysis, **d** AP EUA image in endorotation stress (lateral compression), **e** AP EUA image in exorotation stress (frog position), **f** AP EUA image in push right leg/ pull left leg stress (rotational instability), **g** AP EUA image in push left leg/ pull right leg stress (rotational instability)

The three cases with the greatest percentage change in treatment (#1, #5, and #10) are presented in Figs. 5, 6, and 7.

In case 1 (Fig. 5), the patient had a pelvic binder on during the CT scan. Eventually, 60 surgeons (57%) changed their classification decisions, with a substantial shift observed from the APC1 classification to the APC2 classification (Fig. 1), and 45 surgeons (42%) changed their treatment decisions, with a shift observed from a non-operative treatment choice to both anterior-only or anterior and posterior fixation choices (Fig. 3).

In case 5 (Fig. 6), a patient without a pelvic binder in place during static imaging demonstrated the highest proportion of changes in both classification and treatment, with 82 surgeons (76%) changing their classification, and 84 surgeons (78%) changing their treatment choices. Figure 1 shows a shift from primarily APC1 to both APC2 and APC3, and Fig. 3 shows a shift from a primarily non-operative treatment approach to both anterior-only or anterior and posterior fixation choices.

In case 10 (Fig. 7), a case with no pelvic binder in the static imaging, 52 surgeons (46%) changed classification decisions, while 46 surgeons (43%) changed treatment decisions. This case had almost an equal number of APC 1 and APC2 classifications but shifted to primarily APC 2 after the presentation of EUA images (Fig. 1). Treatment decisions were spread across all three groups for this case, but with the addition of EUA images, the number of surgeons who chose an anterior-only fixation approach increased substantially (Fig. 3).

## Discussion

This study was conducted to assess whether the addition of dynamic stress testing with EUA images influenced surgeons’ classification and treatment decisions for external rotation APC pelvic ring injuries. All cases had some change regarding classification, ranging from 28% to 76%. In those cases, reporting a low incidence of change in classification, the EUA data served primarily as a confirmatory tool for impressions based on static images. However, in those cases reporting higher rates in change of classification, EUA revealed previously unrecognized (occult) pelvic instability. This change in perceived instability was reflected by a concomitant change in more aggressive treatment plans, for which changes ranged from 13% to 78% across all cases.

The substantial variation of the rate of change indicates that EUA serves both as a confirmatory tool in some cases, while detecting occult instability in others. Based on the static images alone, it was not possible to predict which cases would show substantial occult instability or altered management. These findings suggest that EUA adds additional relevant information in a substantial number of patients with APC1 and APC2 pelvic ring injuries when formulating a treatment plan.

The proper classification of APC pelvic ring injuries is a well-known dilemma. The main reason for this is that the classification of these injuries is determined by the ligamentous involvement [1, 19], which is difficult to assess on radiographic images. Pelvic ring injuries are traditionally classified using static radiographs and CT scans; however, these imaging methods have been shown to underestimate instability, potentially due to patient positioning or the use of pelvic binders [3, 4, 6–8, 15]. EUA has been described as a valuable technique used to reveal occult instability [3, 4]. Sagi et al. reported that 50% of the patients initially classified as APC1 were reclassified following EUA, while Suzuki et al. observed a 27% change in classification within the same patient group [3, 4]. Our observed rates of change in classification were consistent with those reported in the literature, with 46% of surgeons changing their classification after the addition of EUA images, predominantly from APC1 to APC2. This study adds to the existing literature by demonstrating the effect of EUA images on surgeons’ interpretation of APC pelvic ring injuries. Although, it should be concluded from this study that even with EUA images, the classification of certain cases remains controversial between surgeons, with variation in classification remaining in some cases. A reason for this may be poor inter-observer reliability for classifying APC injuries. While good inter-observer reliability has been demonstrated for the Young and Burgess classification system as a whole [20, 21], no study has specifically evaluated inter-observer reliability for APC injuries. An additional observation is the shift away from APC3 classification shown in Fig. 2. In theory, if an injury is classified as APC3 on static radiographs, EUA is unnecessary. This shift may be explained by dynamic images appearing more stable than expected, for example, rotational instability being less than 1 cm (Appendix 1), or a limited inter-observer reliability.

Treatment decisions changed in more than a third (36%) of responses in our study, with a shift away from non-operative management towards anterior-only fixation, and minimal change in the choice of combined anterior and posterior treatment approach. Despite the changes in treatment decisions observed, marked differences among surgeons persisted. Previous surveys in the literature have demonstrated similar variability in management preferences. Parry et al. [20] reported substantial inconsistency in the treatment of a single APC3 case, while Moed et al. [21] reported that 56% of surgeons preferred an anterior-only approach for APC2 injuries and 44% opted for combined anterior-posterior fixation. These findings mirror the heterogeneity observed in our survey and may reflect the limited comparative evidence on optimal fixation strategies and postoperative weight-bearing protocols. The variation in decision-making may also relate to surgeon experience, training background, and institutional protocols. Furthermore, the rate of changed treatment decisions varied markedly between cases, ranging from 13 to 78% for patients with APC injuries. This pattern aligns with the results of Tucker et al. [15], who observed a 0–72% change in treatment decisions following EUA in LC-1 injuries. As demonstrated in our study, EUA served as a confirmatory tool in cases with minimal change and revealed occult instability in cases with greater change. Like our design, their survey included 10 cases, but only 11 surgeons were surveyed. Our findings extend this evidence to APC injuries and represent one of the first investigations of how EUA influences both classification and treatment decisions in this specific injury group.

An important clinical question to consider is whether EUA should be performed for all suspected APC injuries. Our findings suggest that EUA is useful when static imaging indicates relative stability and nonoperative management is being considered, as occult instability may alter treatment. Therefore, when possible, EUA should be performed in clinical practice.

Some limitations of this study should be mentioned. First, although 400 surgeons were invited, only 123 responded, giving a response rate of 31%. While this is less than desired, it compares favorably with other large international surveys, reporting an 8% response rate [21]. Moreover, the respondents represented an international cohort of experienced pelvic surgeons, with an average of 10 years of experience treating pelvic ring injuries and approximately 50 pelvic ring injuries treated per year per surgeon, making the group representative of expert clinical practice. However, a substantial proportion of respondents originated from Germany, Italy, and the Netherlands. Differences in healthcare systems, surgical training, and regional treatment protocols may influence management, which could affect the generalizability of the findings. Country-specific differences should be considered when interpreting the results. Secondly, the survey included 10 cases selected by pelvic surgeons with over 10 years of experience to illustrate a range of APC injury patterns. While this selection may not cover the entire spectrum of APC injuries, the observed variability in responses suggests that a broad range of injury severities was represented.

Additionally, this study does not allow assessment of the correctness or accuracy of classification or treatment decisions; rather, it evaluates changes in classification and treatment recommendations following the addition of EUA images. Also, no interobserver reliability is known for this method. Furthermore, approximately half of the CT scans included pelvic binders, which could be viewed as a limitation. However, this was intentional to replicate real-world conditions in level 1 trauma centers [16], and changes in classification and treatment decisions were observed in both binder and non-binder cases. Another potential limitation is that surgeons who routinely perform EUA may interpret findings differently from those who do not, or those treating more than 50 pelvic injuries per year compared to fewer than 50; however, this distinction also reflects clinical practice. A sub-analysis comparing these groups revealed few significant differences in classification or treatment decision changes.

## Conclusions

This survey indicates that the addition of EUA images alters surgeons’ perception of the injury. A total rate of change of 46% was observed for classification, resulting in a change in treatment plan in 36% of the cases analyzed. The greatest shifts occurred with an APC1 classification being changed to APC2, and non-operative treatment changing to anterior only fixation. These findings show that dynamic stress-testing with EUA and fluoroscopy provides additional information with respect to perceived instability in pelvic ring injuries, with partial instability leading to a change in treatment plan in a substantial proportion of patients.

## Supplementary Information

Below is the link to the electronic supplementary material.

Supplementary Material 1

Supplementary Material 2

Supplementary Material 3

Supplementary Material 4

Supplementary Material 5

Supplementary Material 6

## Data Availability

No datasets were generated or analysed during the current study.
