# Supplementary material for: Examination under anesthesia imaging changes surgeons’ classification and treatment decisions of anterior posterior compression pelvic ring injuries
Source: Eur J Orthop Surg Traumatol. 2026 May 30;36(1):202. doi: 10.1007/s00590-026-04744-8 (PMC13222183; doi:10.1007/s00590-026-04744-8)
Supplement: Supplementary file 1 — Supplementary Material 1 [file 590_2026_4744_MOESM1_ESM.docx]

**Appendix 1** Proposed EUA measurements and resulting treatments from the article by Sagi et al. (2)

| EUA measurements | Injury characteristics | Treatment |
| --- | --- | --- |
| <2.5 cm diastasis (exorotation) | Intact sacroiliac ligaments | Non-operative treatment |
| <2.5 cm diastasis (exorotation) | Disrupted anterior sacroiliac ligaments | Add anterior pelvic ring fixation |
| >1cm rotational instability | Disrupted posterior sacroiliac ligaments | Add posterior pelvic ring fixation |
