# Supplementary material for: Examination under anesthesia imaging changes surgeons’ classification and treatment decisions of anterior posterior compression pelvic ring injuries
Source: Eur J Orthop Surg Traumatol. 2026 May 30;36(1):202. doi: 10.1007/s00590-026-04744-8 (PMC13222183; doi:10.1007/s00590-026-04744-8)
Supplement: Supplementary file 2 — Supplementary Material 2 [file 590_2026_4744_MOESM2_ESM.docx]

**Appendix 2** Cases presented in the EUA survey, including static and dynamic images.

| Case 1 | |
| --- | --- |
| AP Radiograph: | 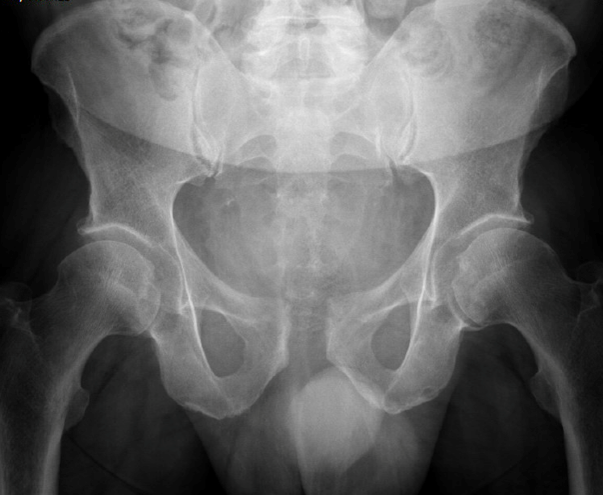 |
| Axial CT Scan:  * Double-click the CT image to view the video | **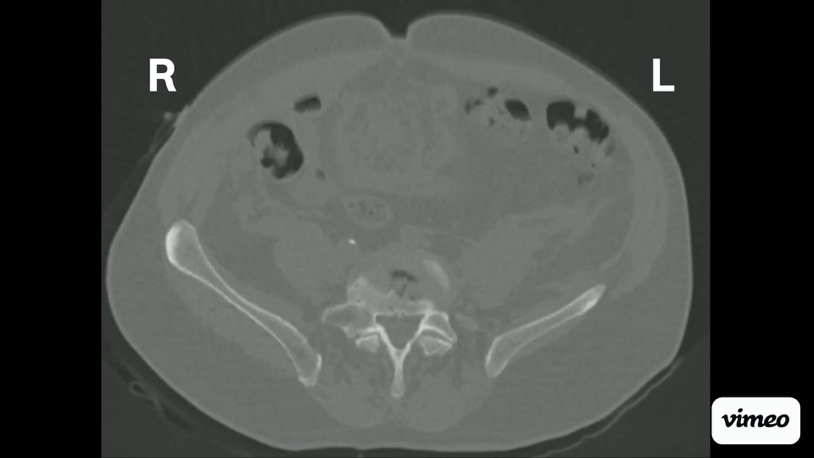** |
| Endorotation Stress (lateral compression): | 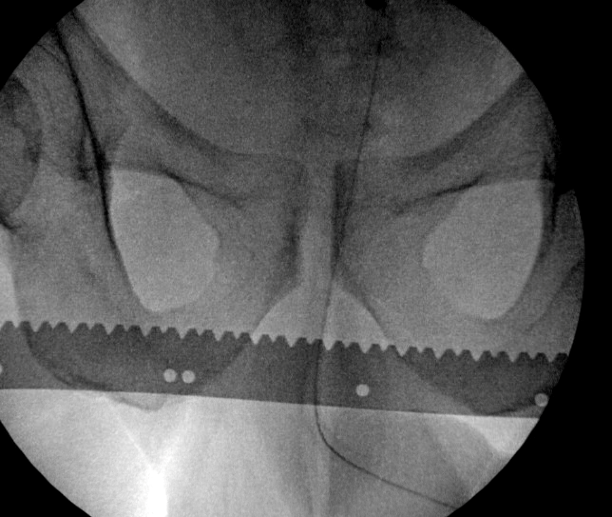 |
| Exorotation Stress (frog position): | 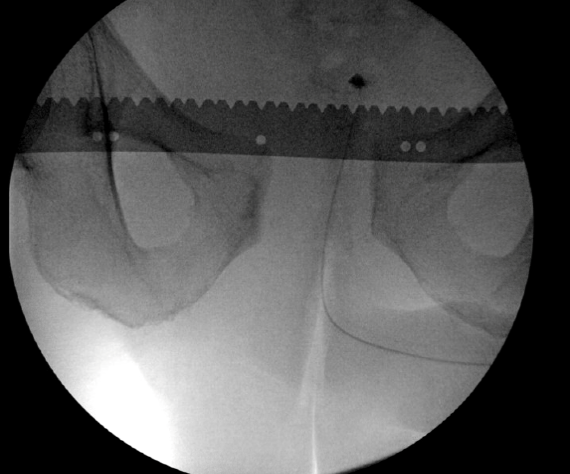 |
| Push right leg/ pull left leg: | 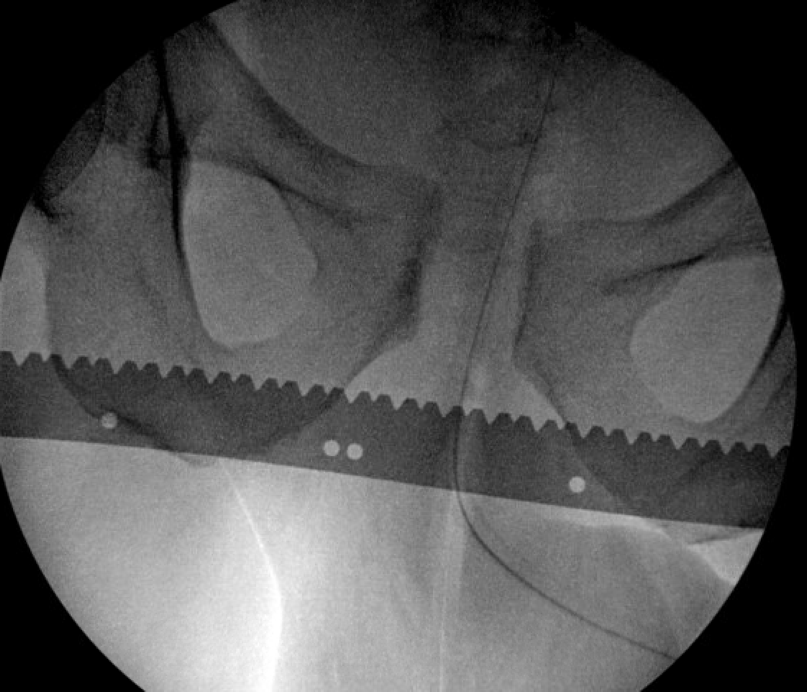 |
| Push left leg/ pull right leg: | 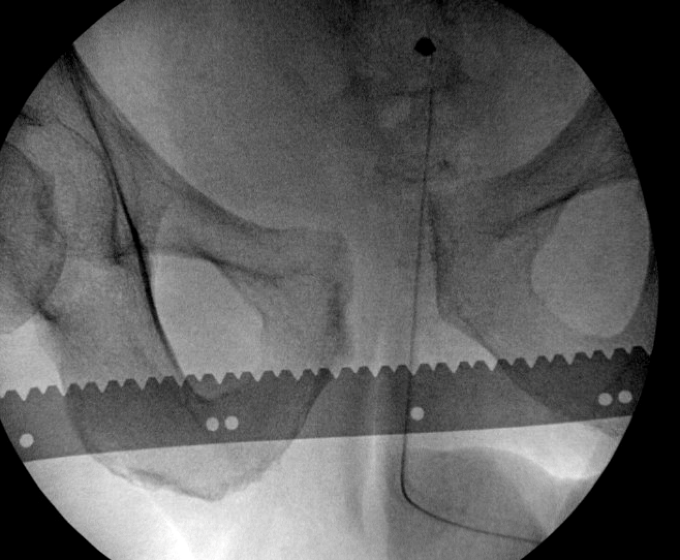 |

| Case 2 | |
| --- | --- |
| AP Radiograph: | 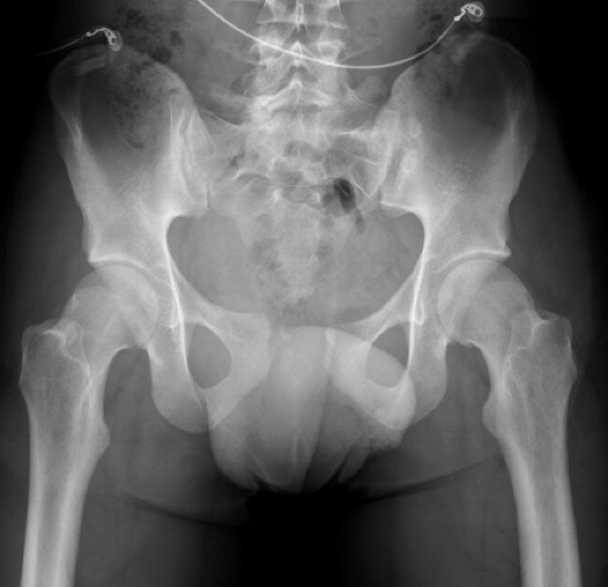 |
| Axial CT Scan:  * Double-click the CT image to view the video | 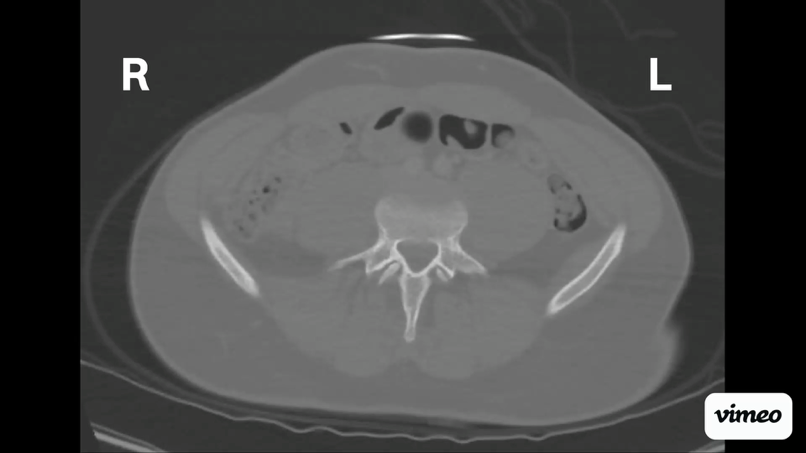 |
| Endorotation Stress (lateral compression): | 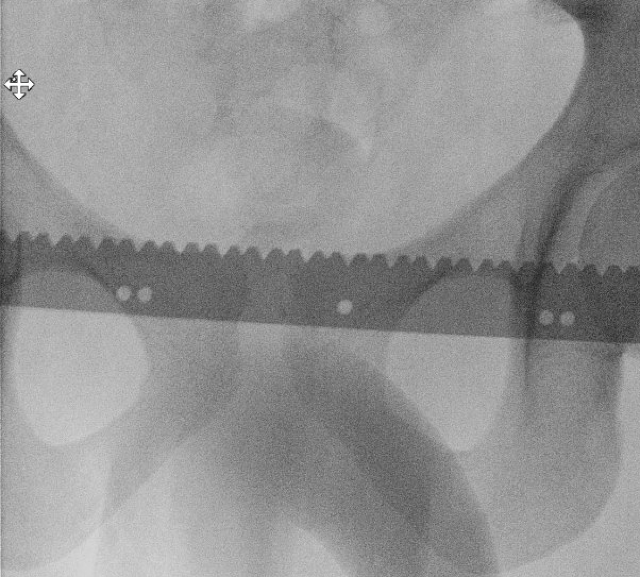 |
| Exorotation Stress (frog position): | 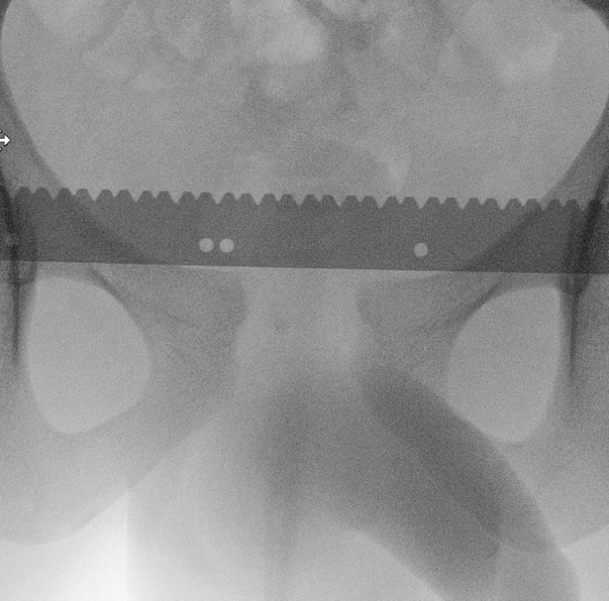 |
| Push right leg/ pull left leg: | 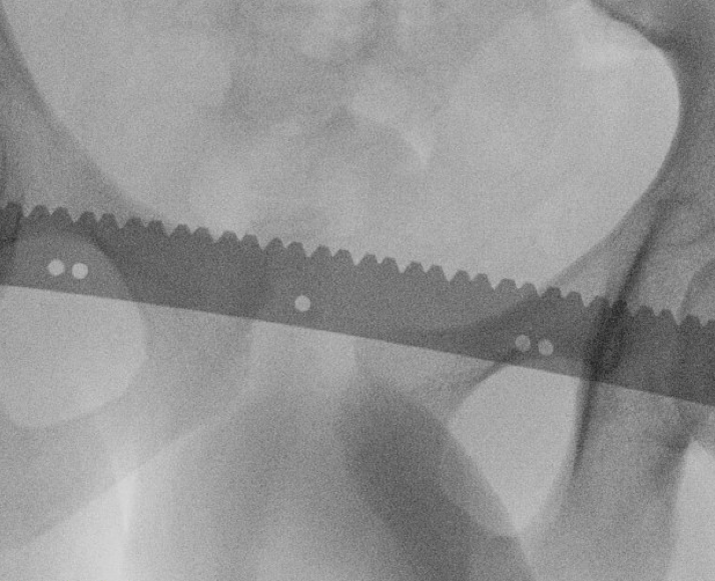 |
| Push left leg/ pull right leg: | 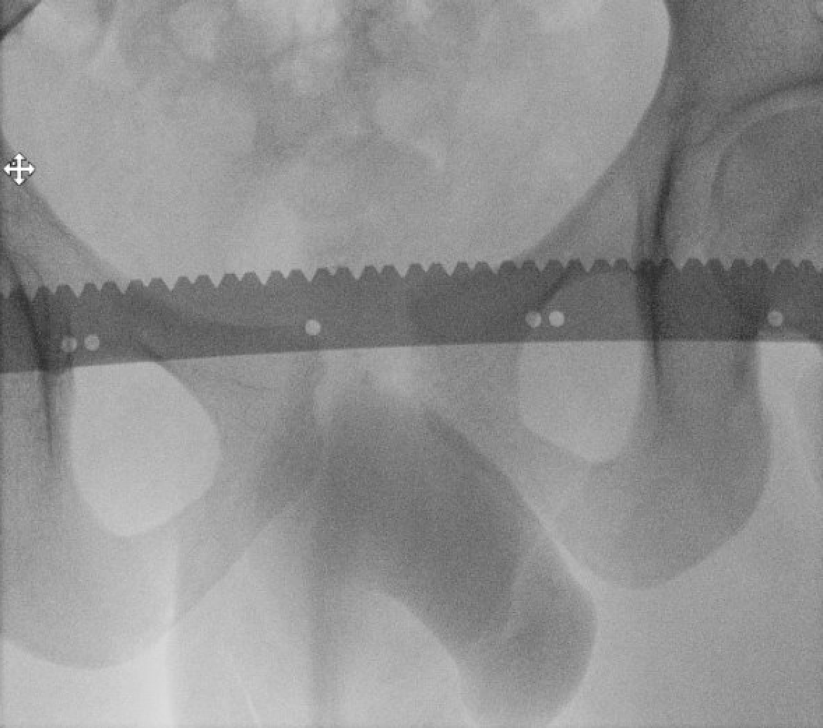 |

| Case 3 | |
| --- | --- |
| AP Radiograph: | 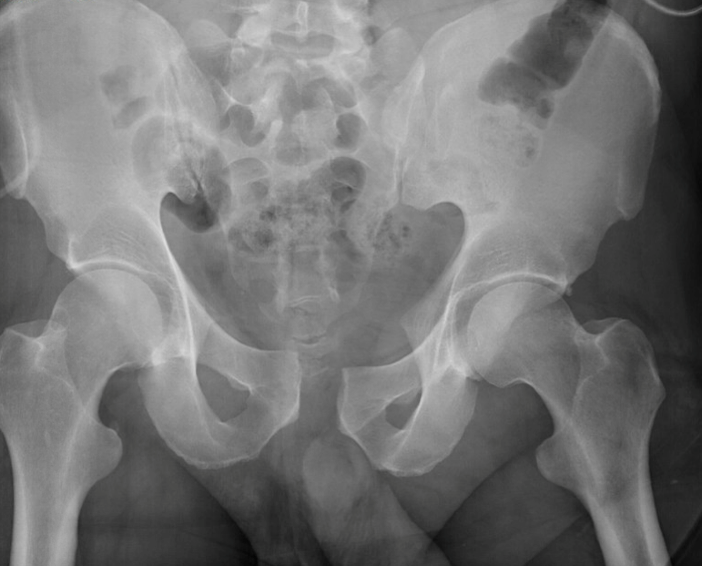 |
| Axial CT Scan:  * Double-click the CT image to view the video | 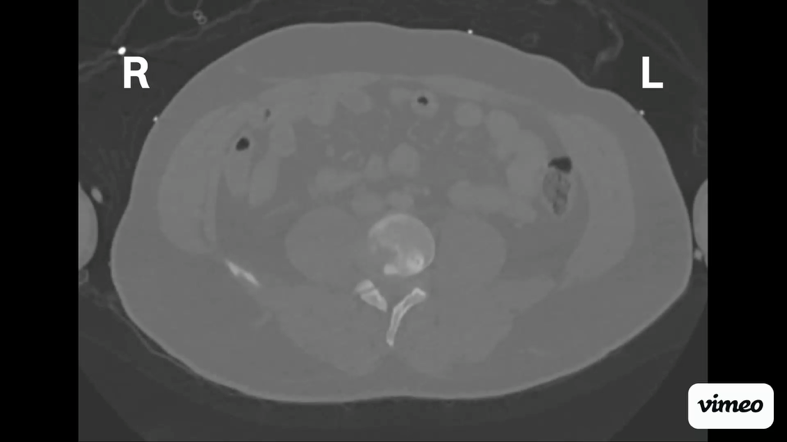 |
| Endorotation Stress (lateral compression): | 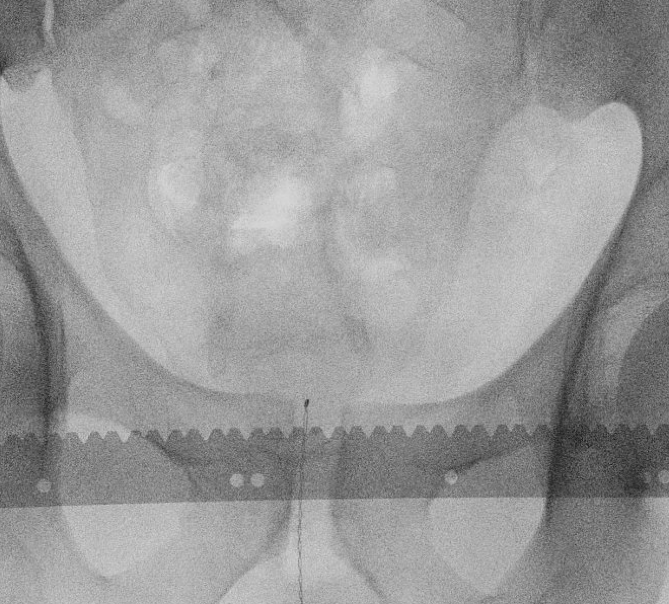 |
| Exorotation Stress (frog position): | 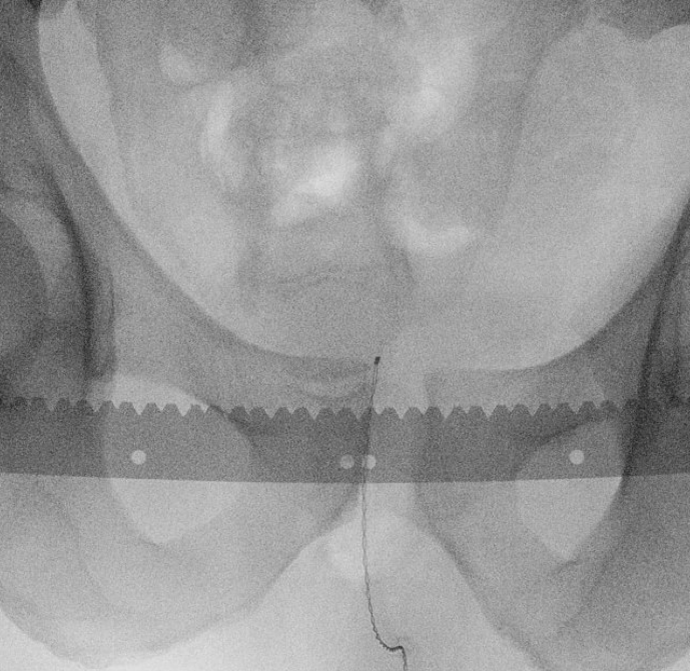 |
| Push right leg/ pull left leg: | 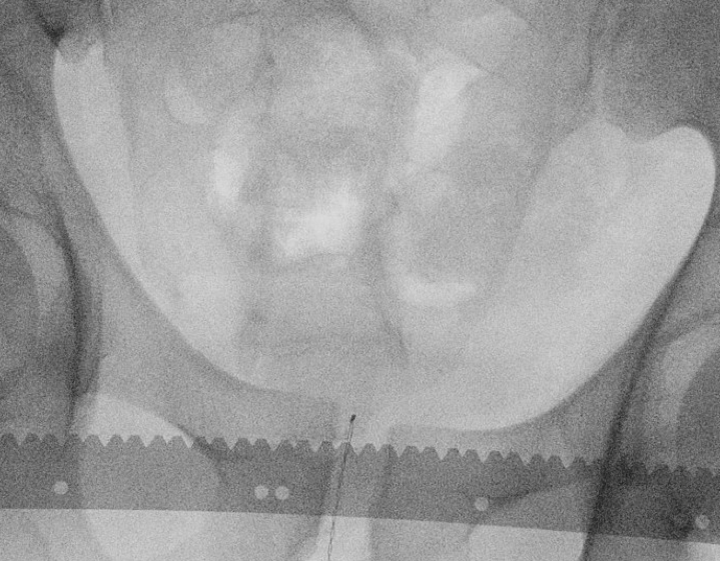 |
| Push left leg/ pull right leg: | 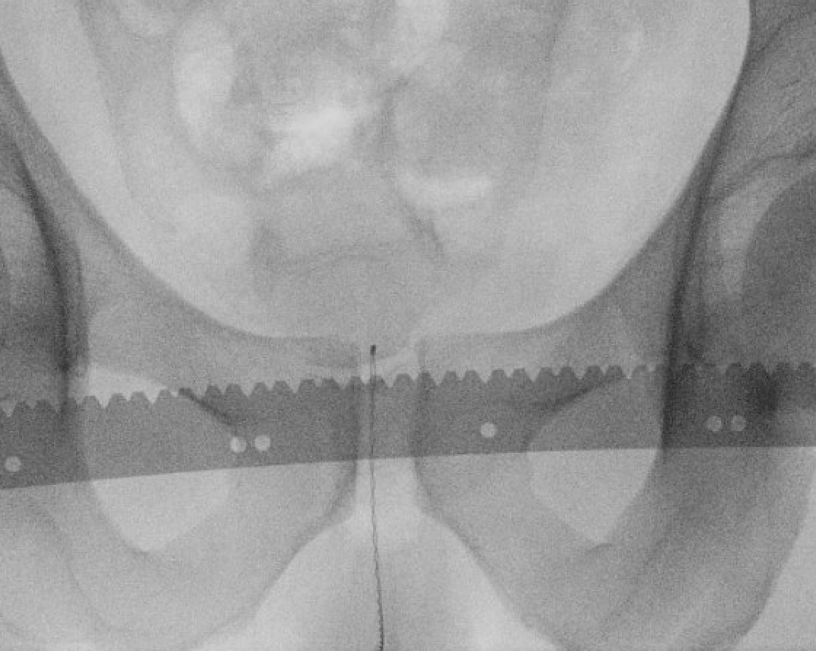 |

| Case 4 | |
| --- | --- |
| AP Radiograph: | 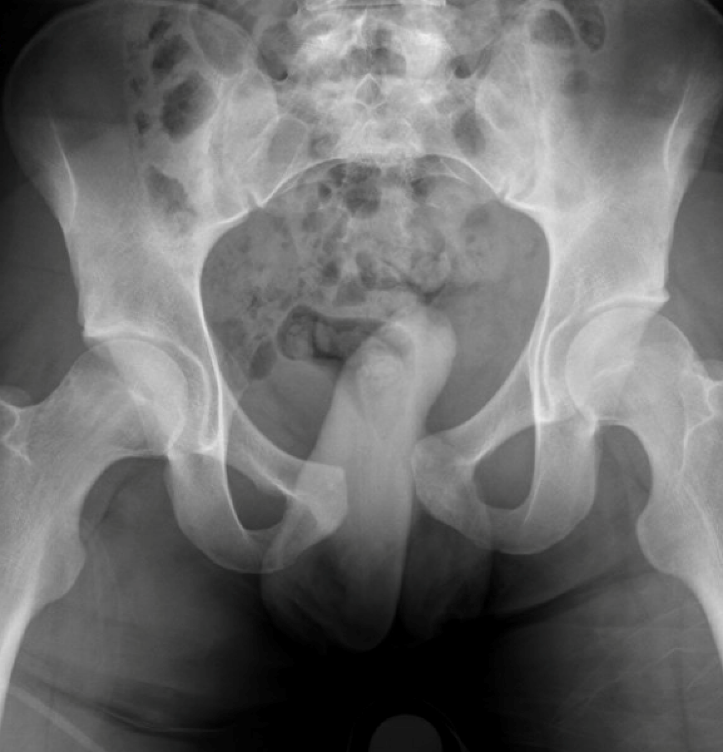 |
| Axial CT Scan:  * Double-click the CT image to view the video | 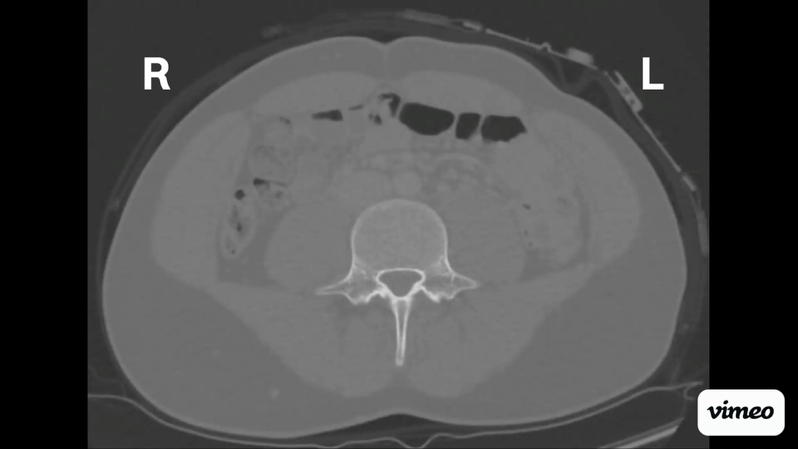 |
| Endorotation Stress (lateral compression): | 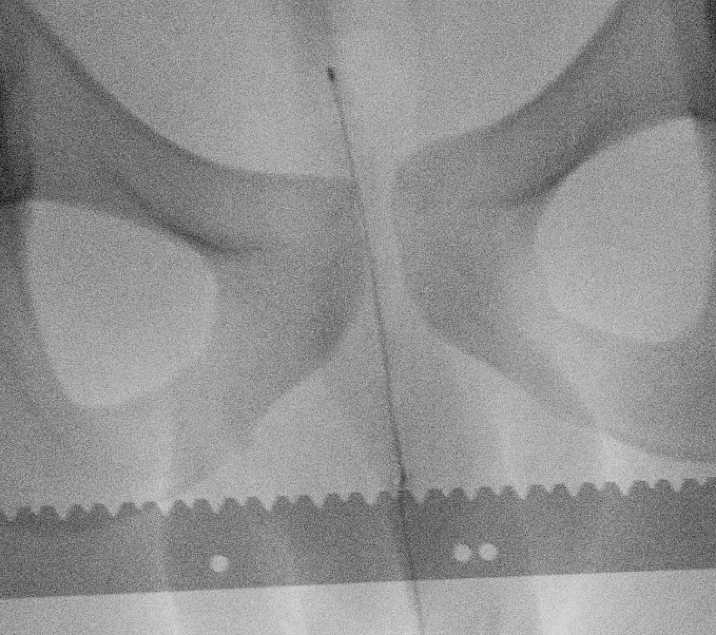 |
| Exorotation Stress (frog position): | 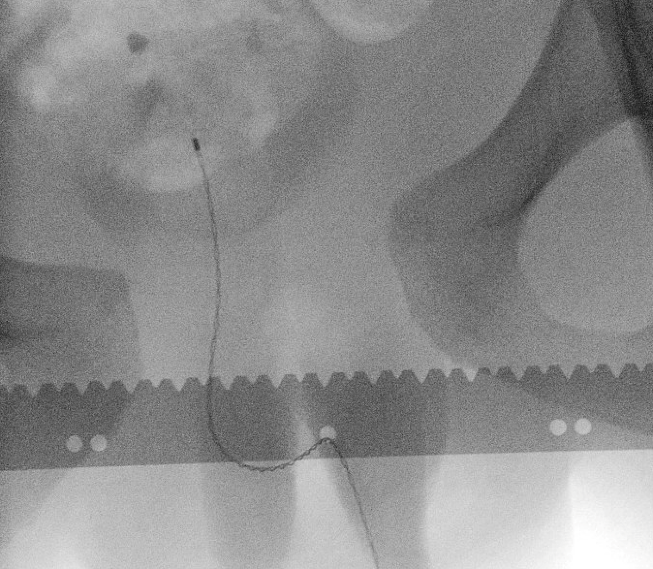 |
| Push right leg/ pull left leg: | 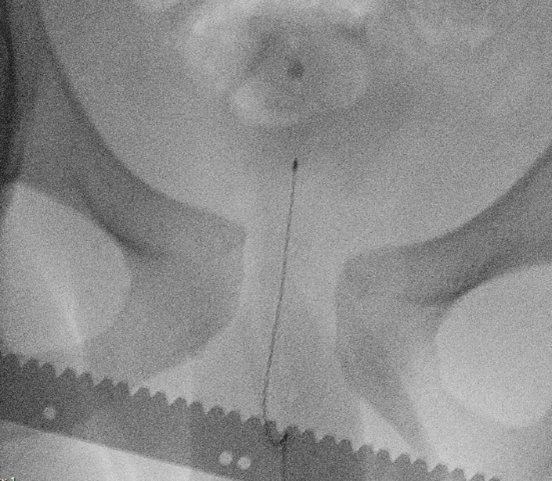 |
| Push left leg/ pull right leg: | 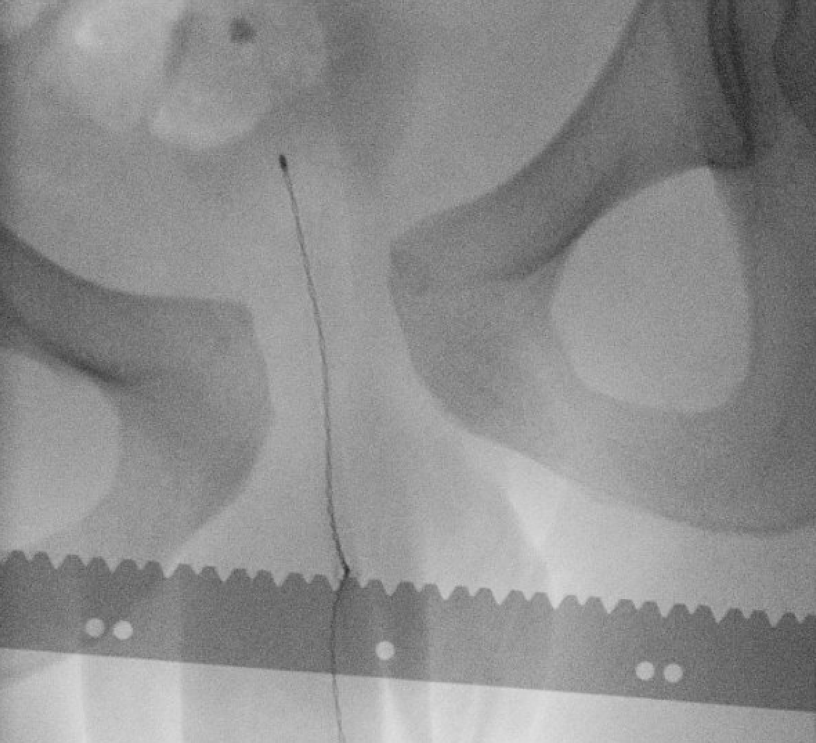 |

| Case 5 | |
| --- | --- |
| AP Radiograph: | 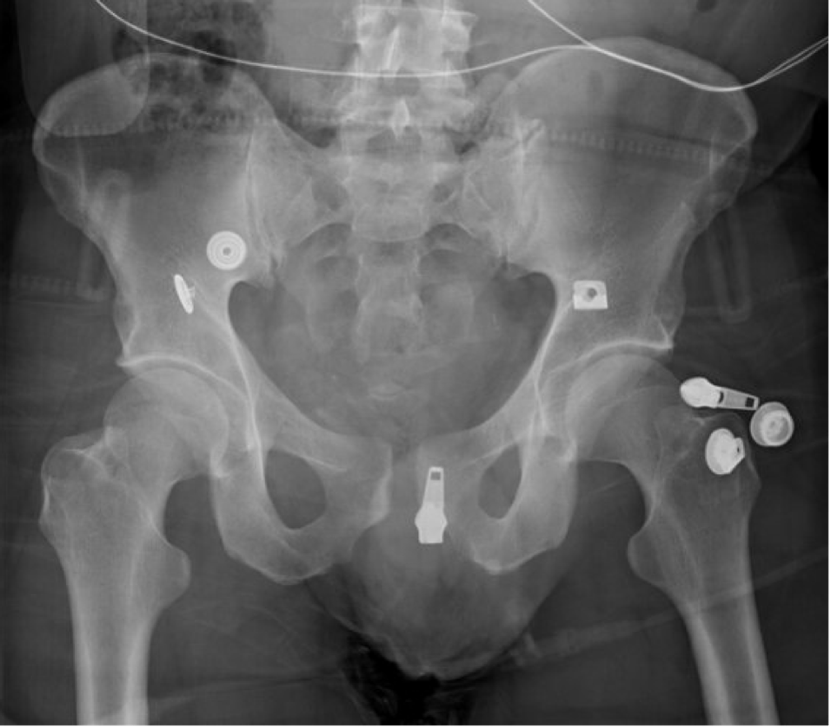 |
| Axial CT Scan:  * Double-click the CT image to view the video | 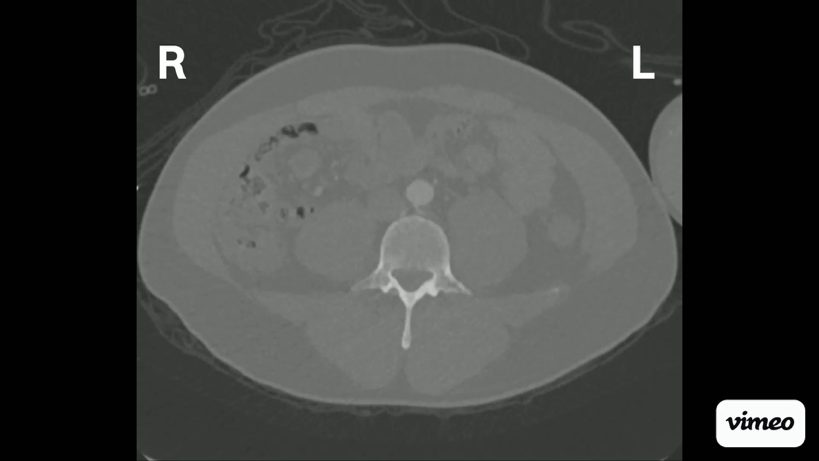 |
| Endorotation Stress (lateral compression): | 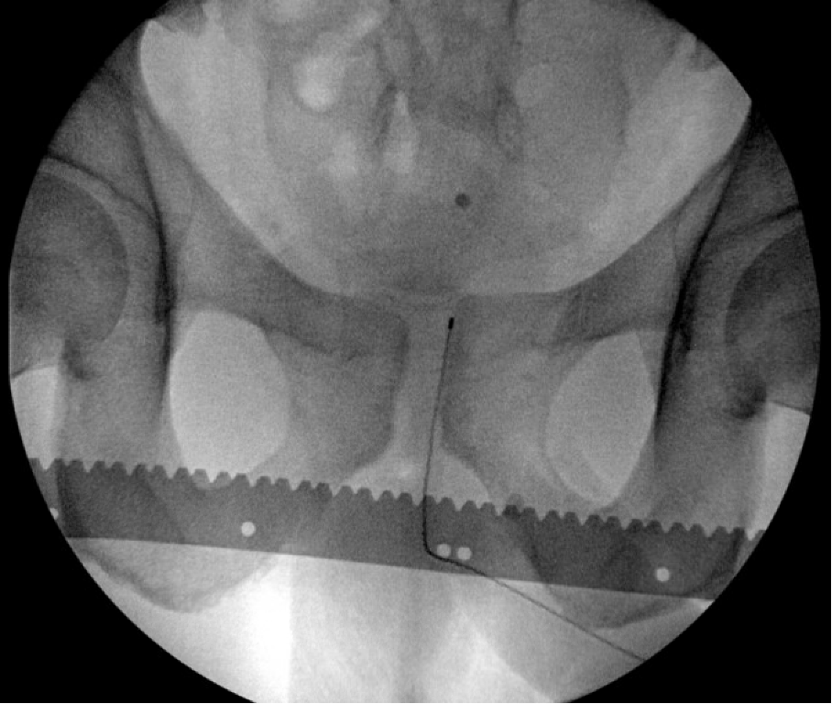 |
| Exorotation Stress (frog position): | 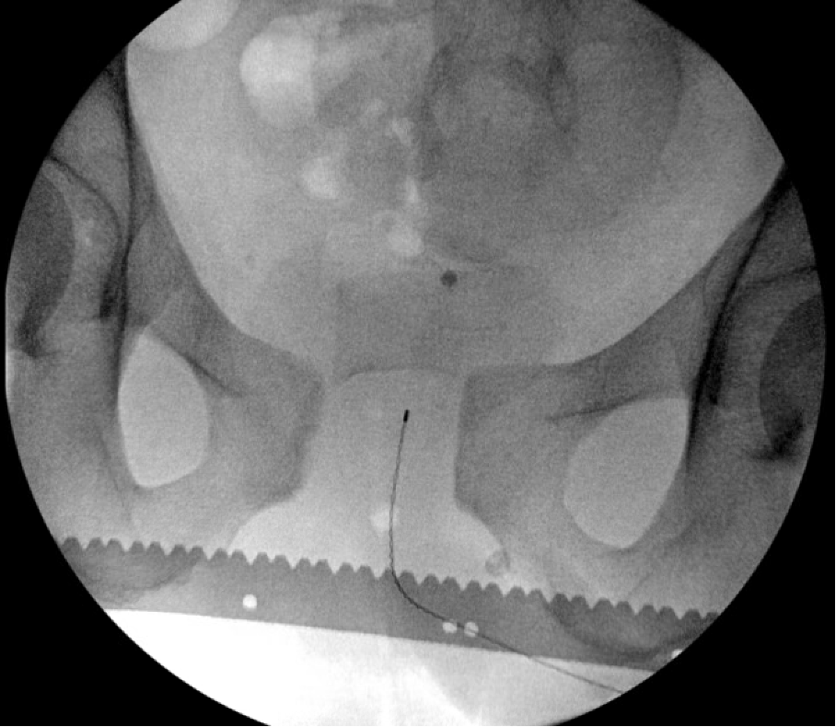 |
| Push right leg/ pull left leg: | 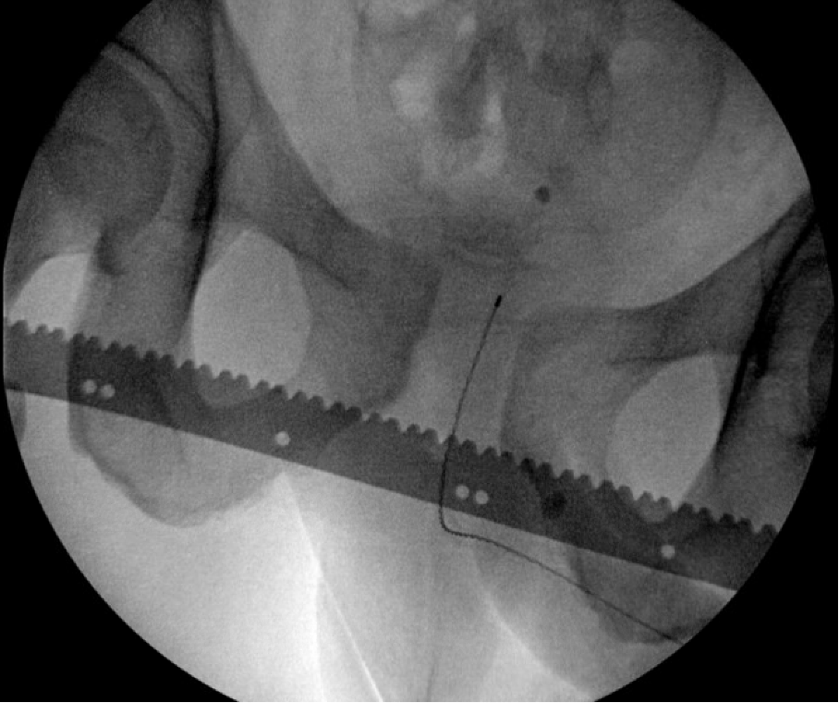 |
| Push left leg/ pull right leg: | 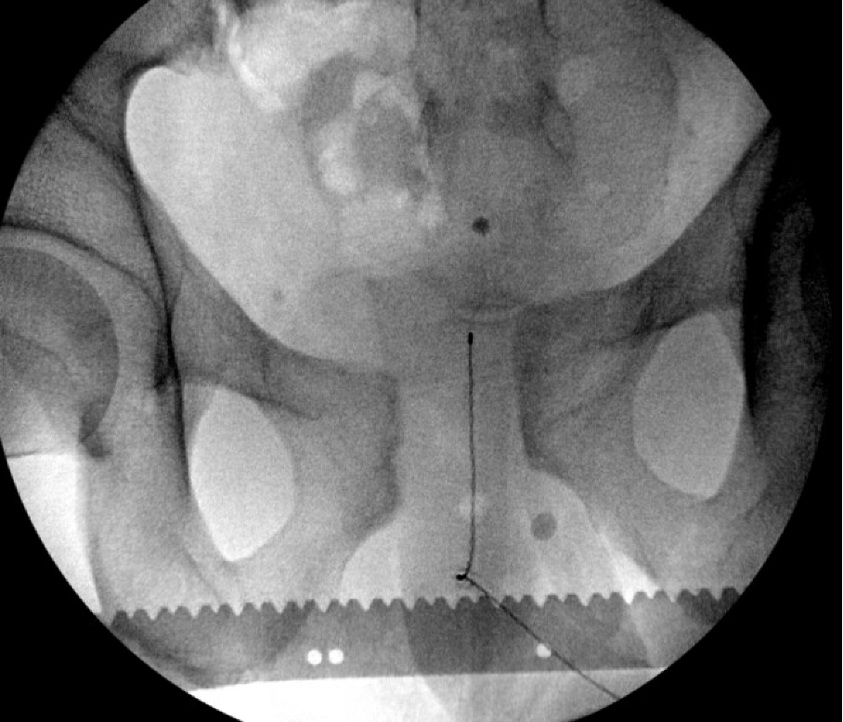 |

| Case 6 | |
| --- | --- |
| AP Radiograph: | 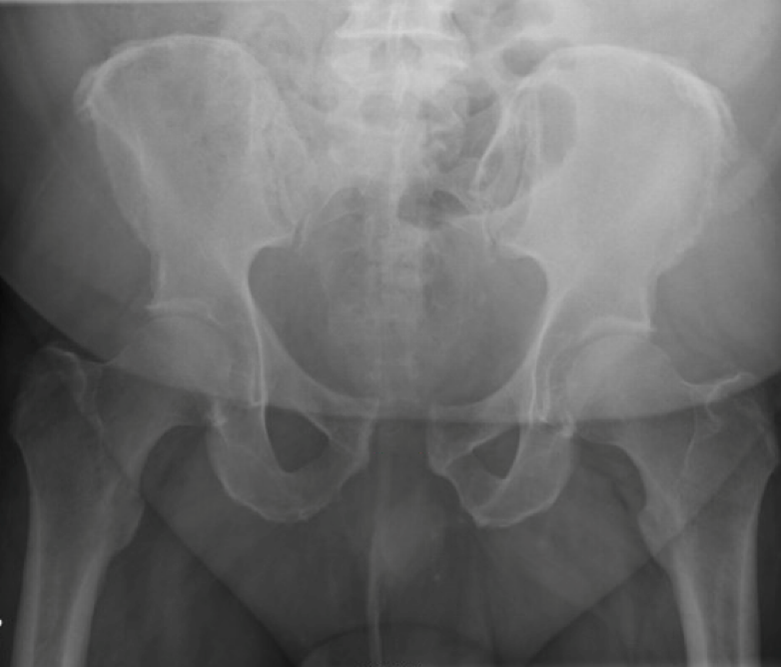 |
| Axial CT Scan:  * Double-click the CT image to view the video | 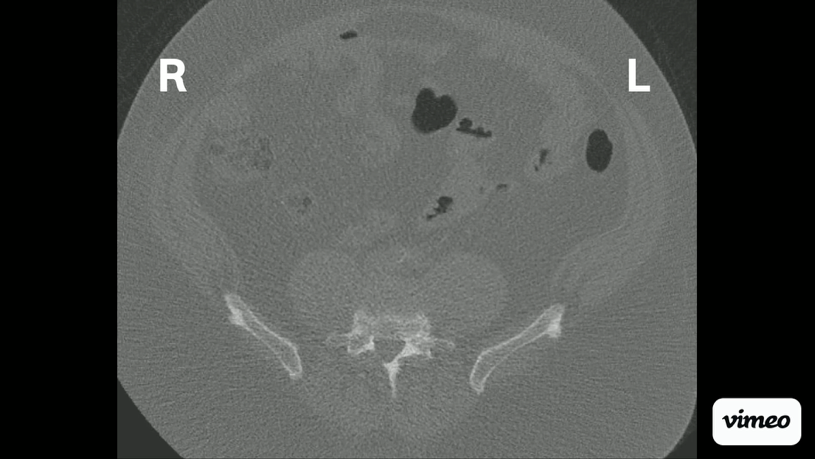 |
| Endorotation Stress (lateral compression): | 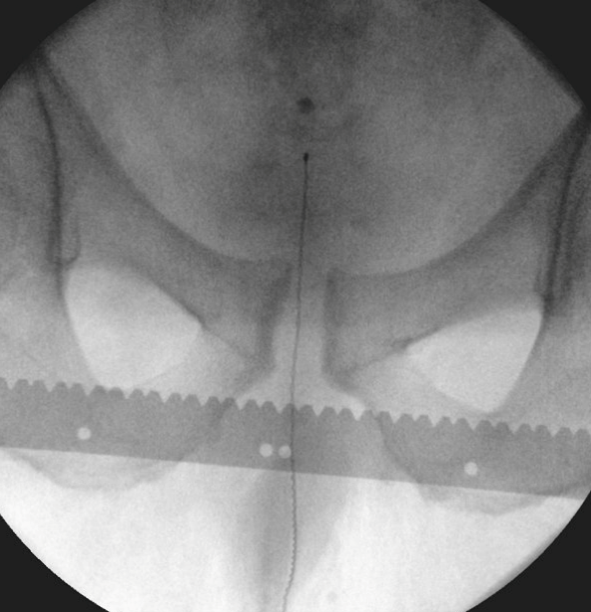 |
| Exorotation Stress (frog position): | 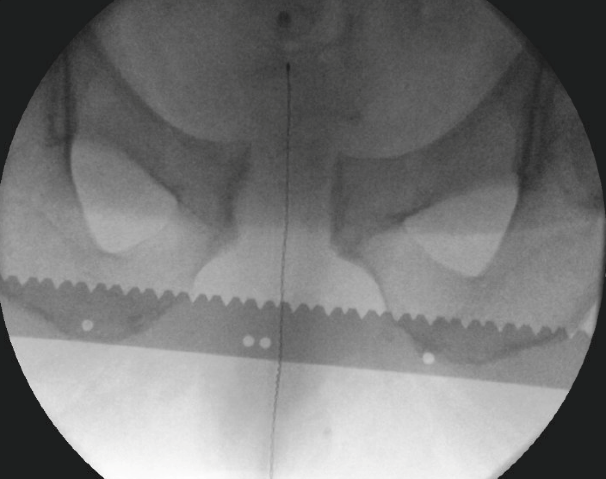 |
| Push right leg/ pull left leg: | 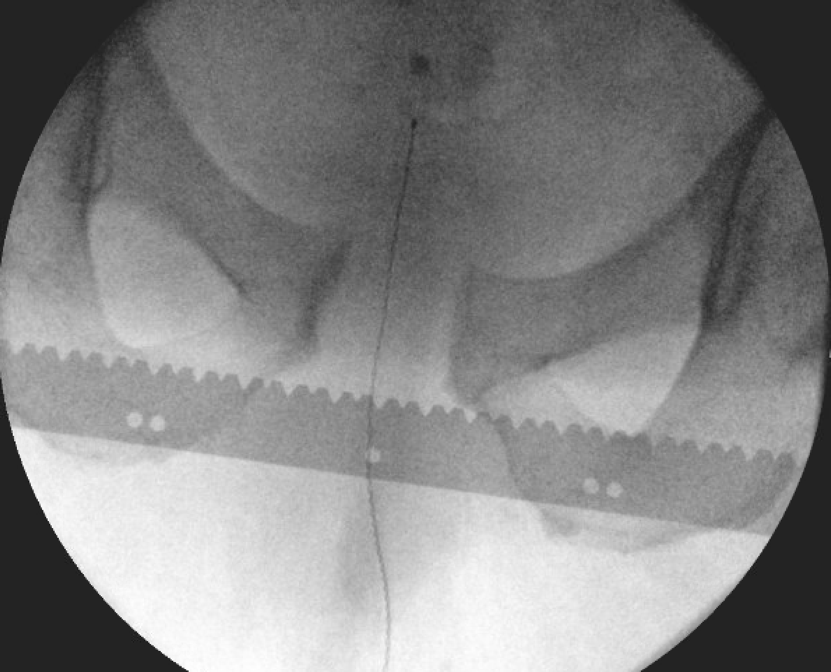 |
| Push left leg/ pull right leg: | 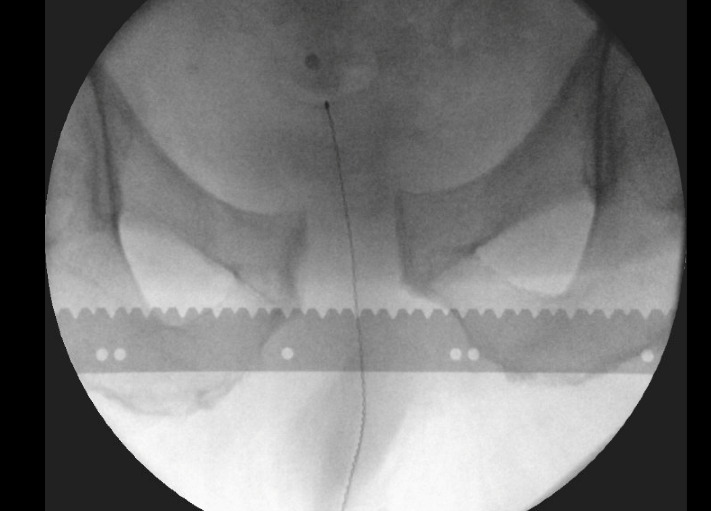 |

| Case 7 | |
| --- | --- |
| AP Radiograph: | 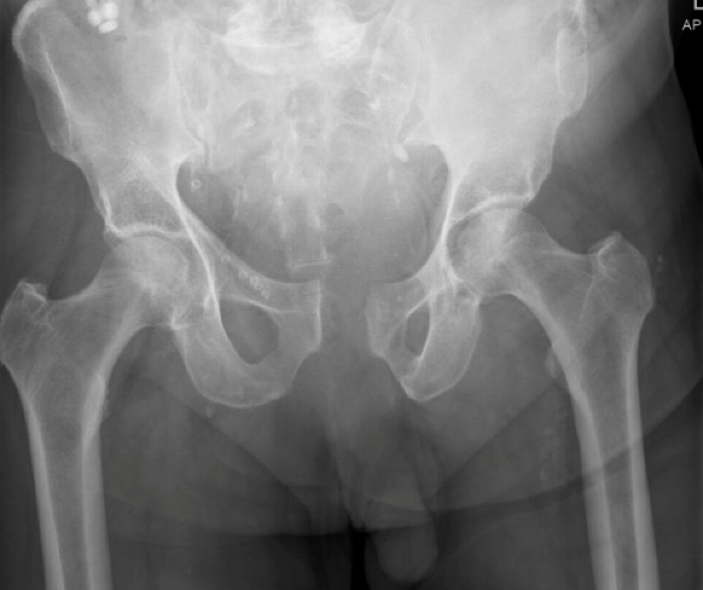 |
| Axial CT Scan:  * Double-click the CT image to view the video | 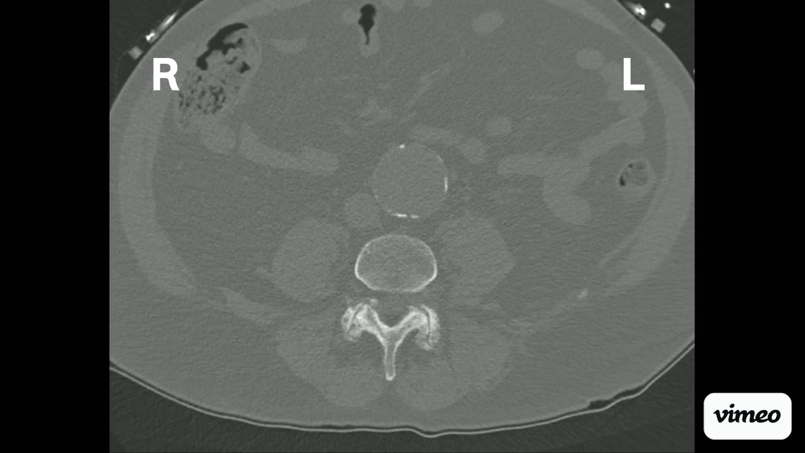 |
| Endorotation Stress (lateral compression): | 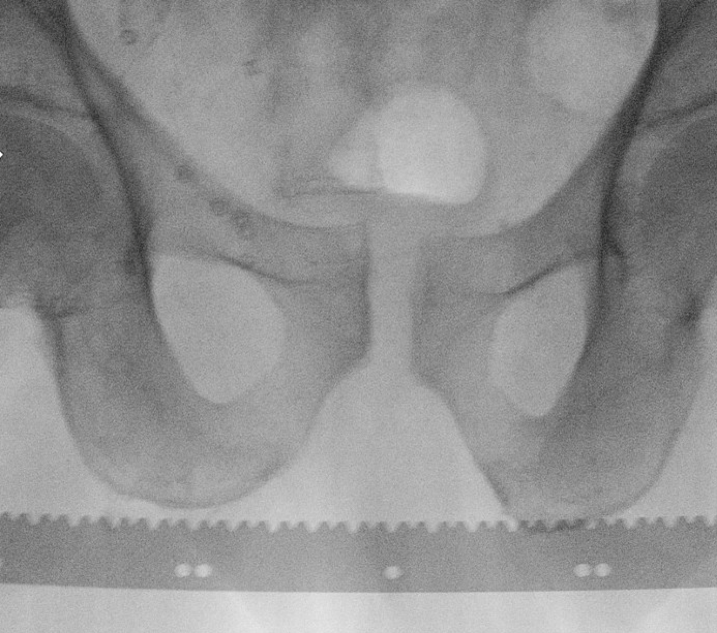 |
| Exorotation Stress (frog position): | 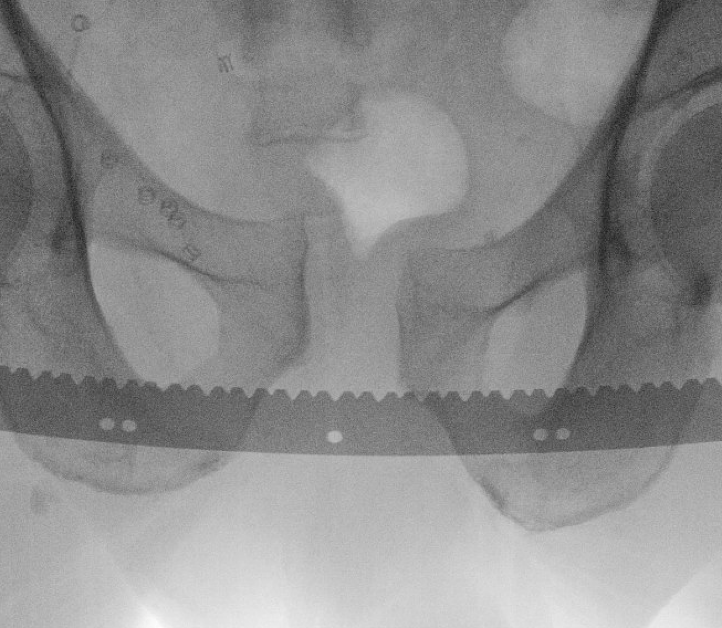 |
| Push right leg/ pull left leg: | 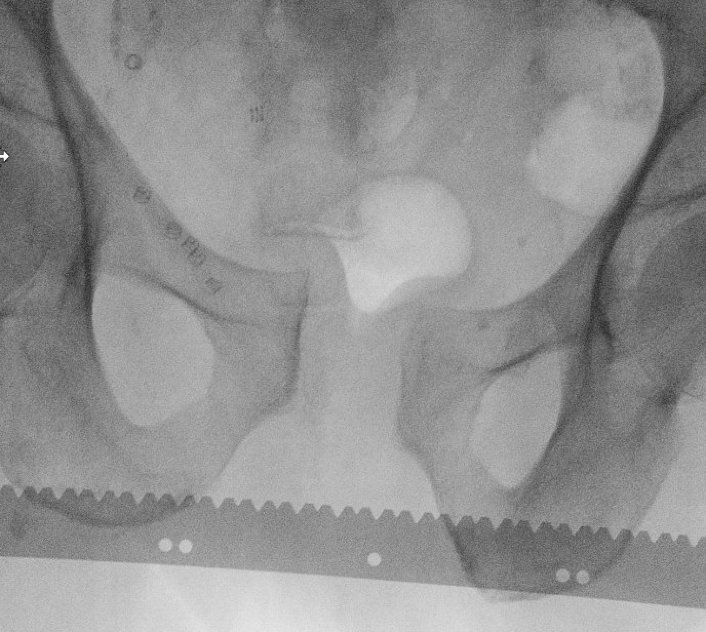 |
| Push left leg/ pull right leg: | 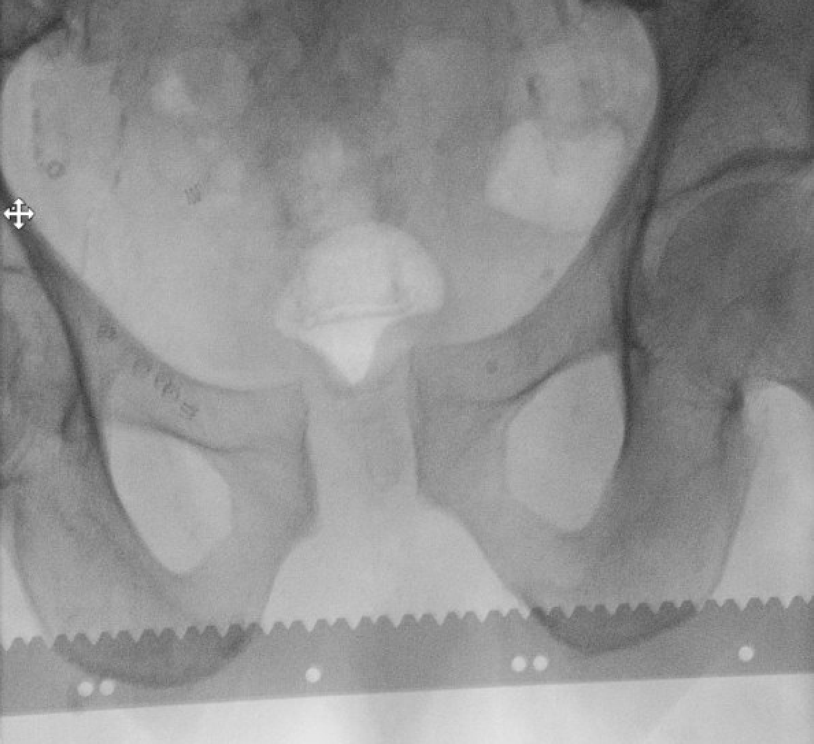 |

| Case 8 | |
| --- | --- |
| AP Radiograph: | 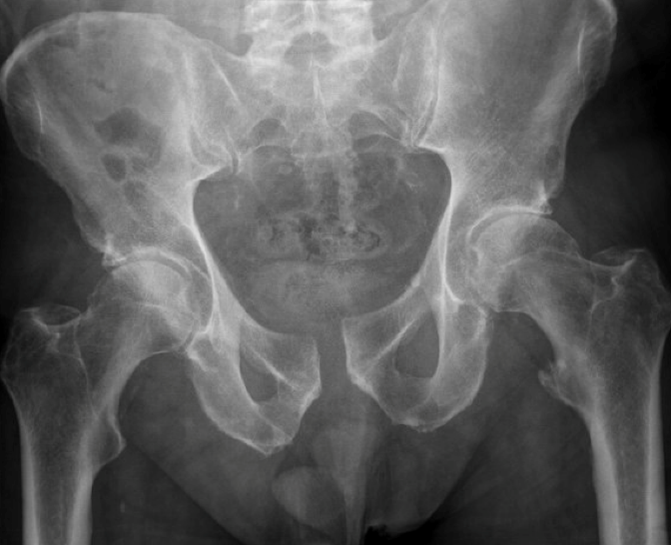 |
| Axial CT Scan:  * Double-click the CT image to view the video | 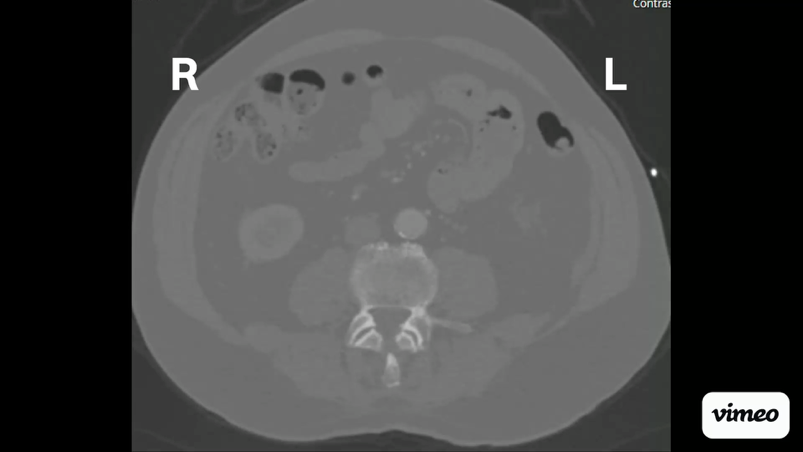 |
| Endorotation Stress (lateral compression): | 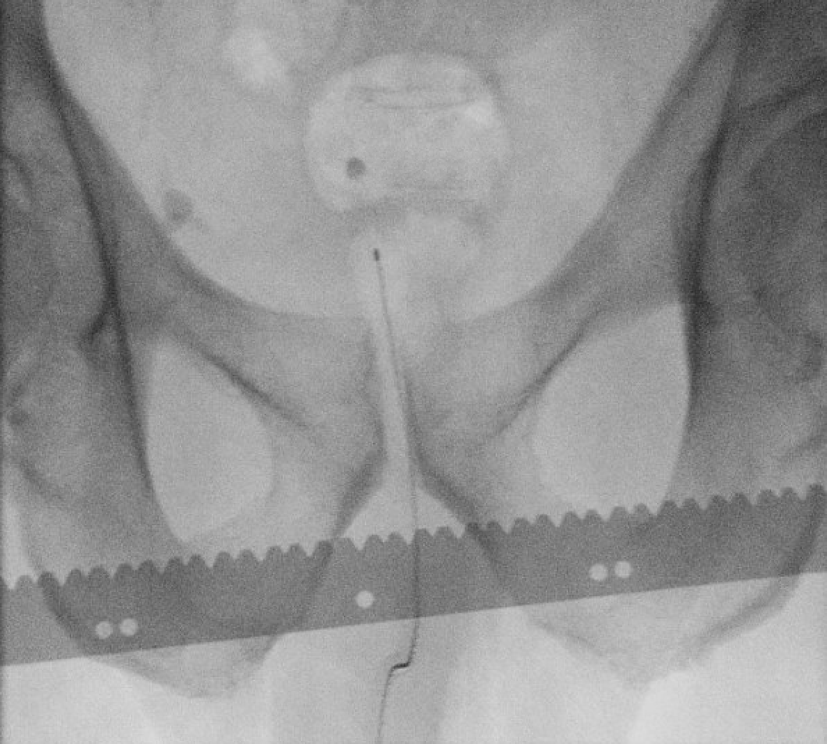 |
| Exorotation Stress (frog position): | 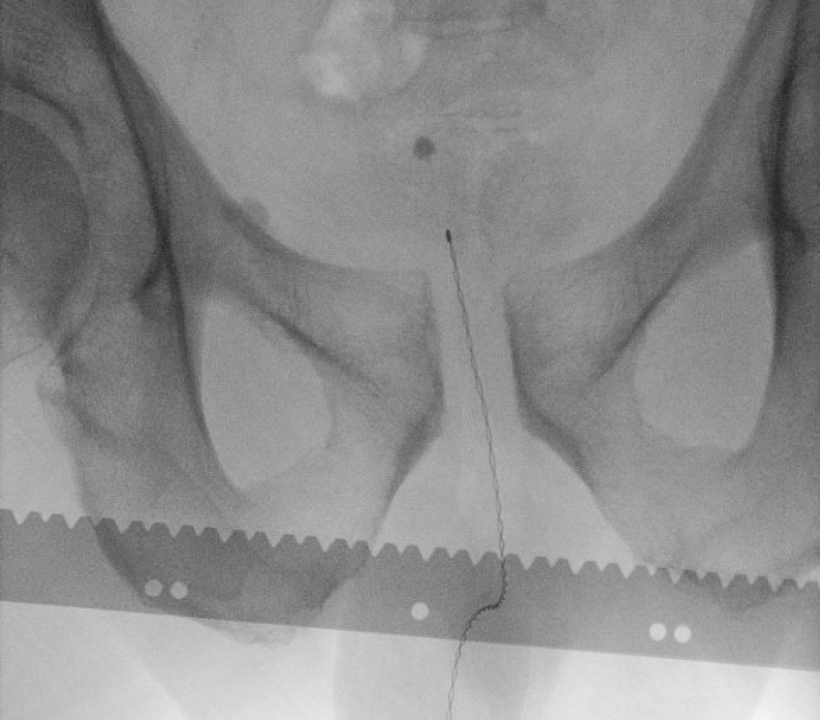 |
| Push right leg/ pull left leg: | 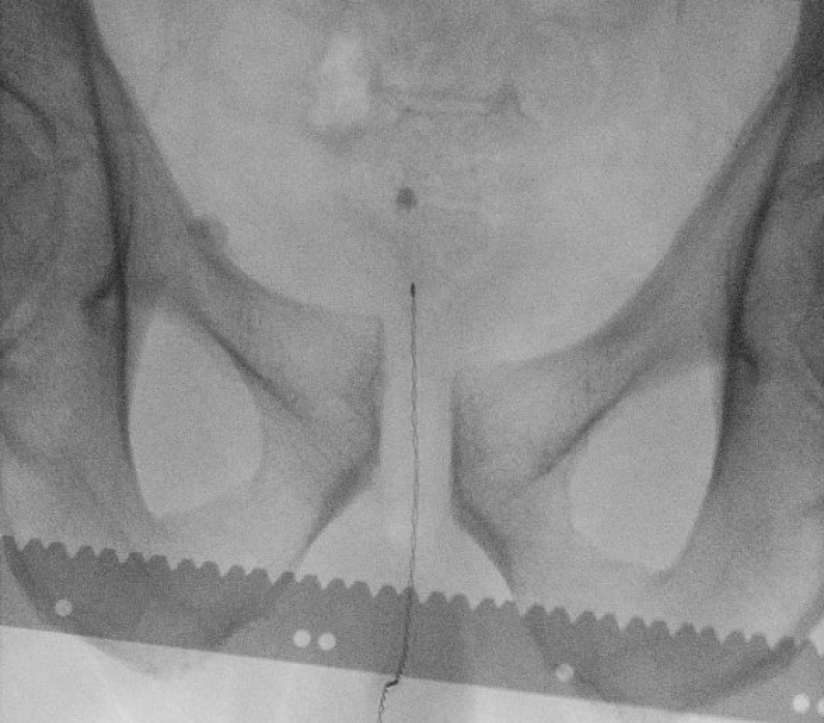 |
| Push left leg/ pull right leg: | 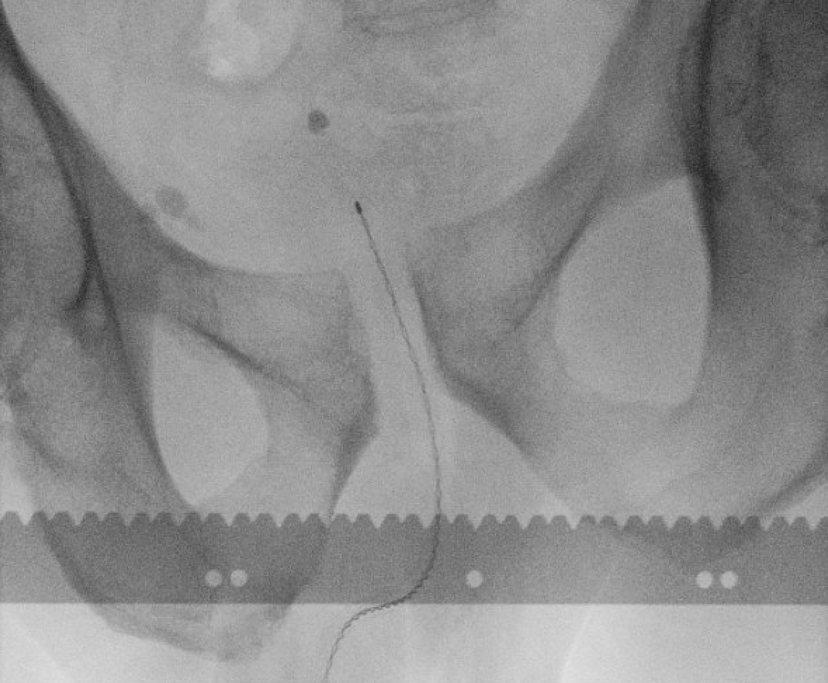 |

| Case 9 | |
| --- | --- |
| AP Radiograph: | 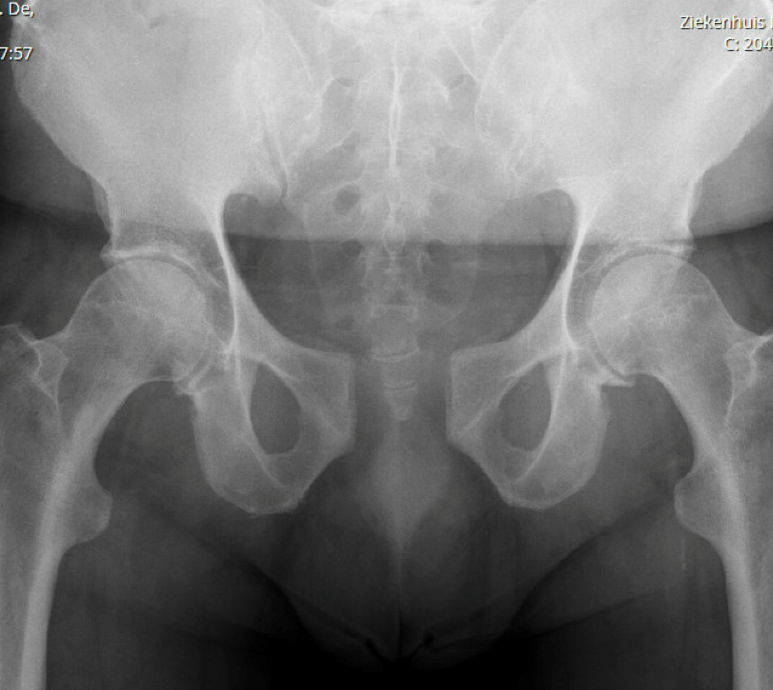 |
| Axial CT Scan:  * Double-click the CT image to view the video | 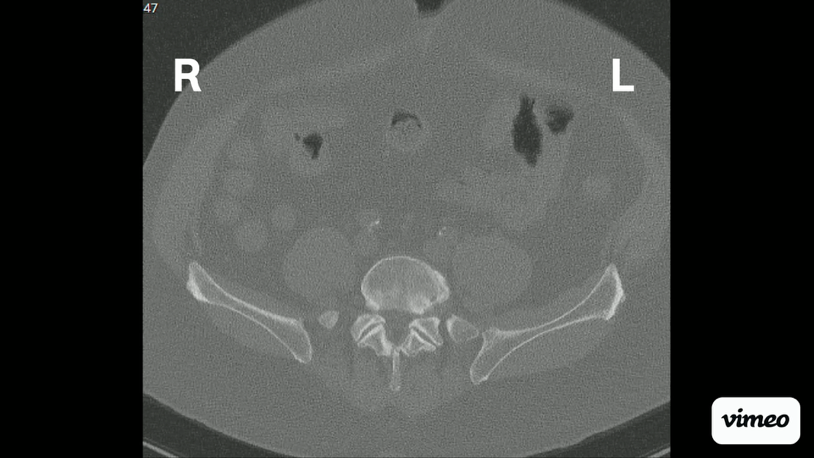 |
| Endorotation Stress (lateral compression): | 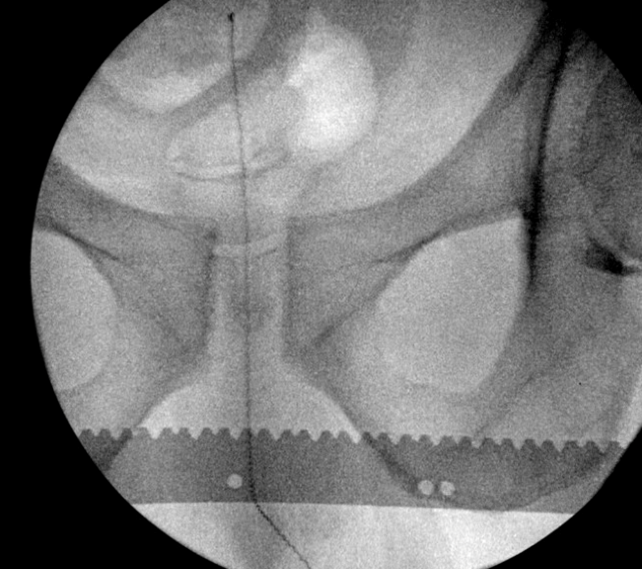 |
| Exorotation Stress (frog position): | 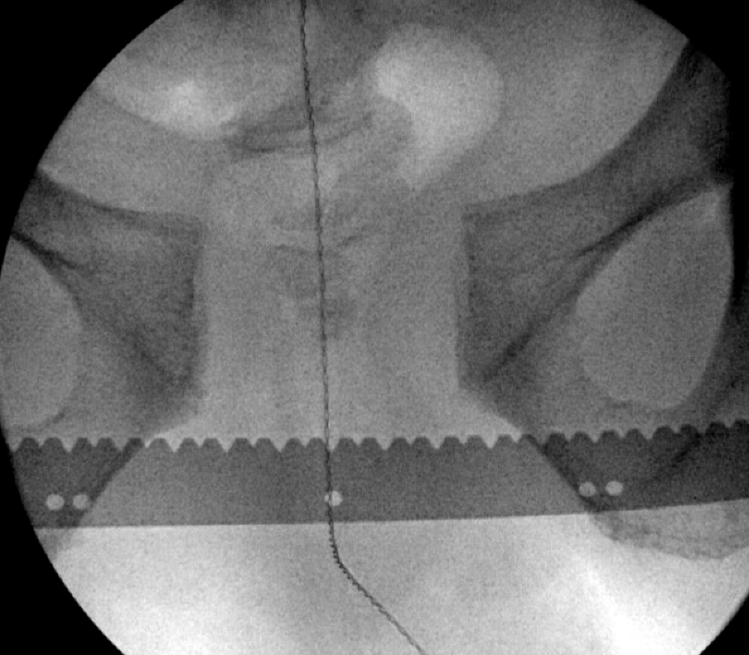 |
| Push right leg/ pull left leg: | 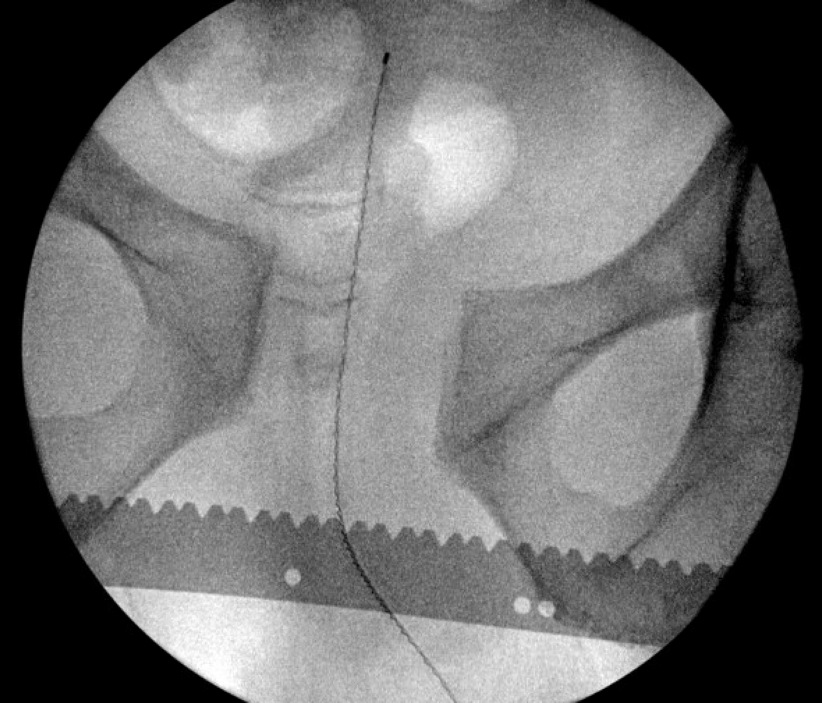 |
| Push left leg/ pull right leg: | 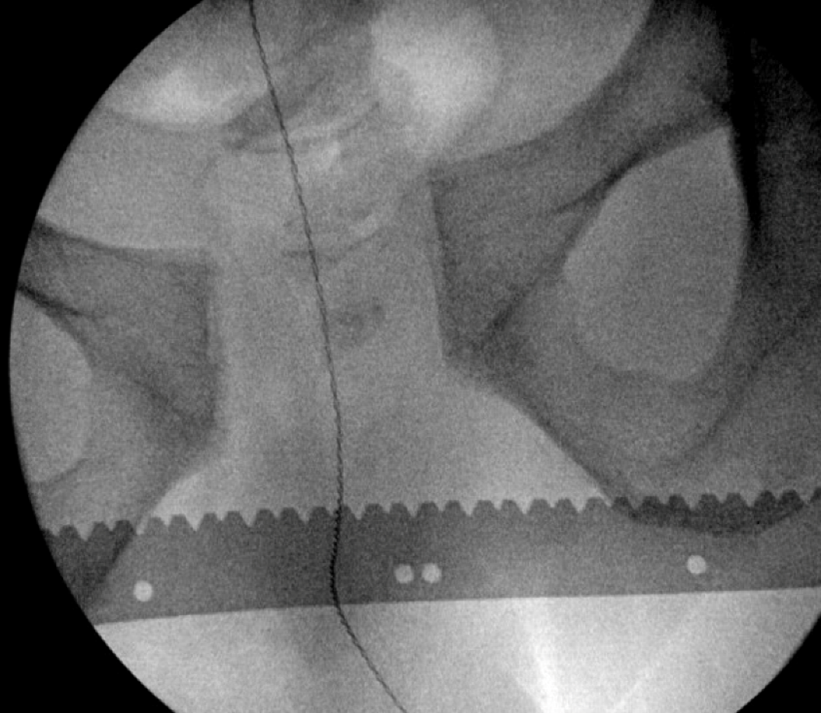 |

| Case 10 | |
| --- | --- |
| AP Radiograph: | 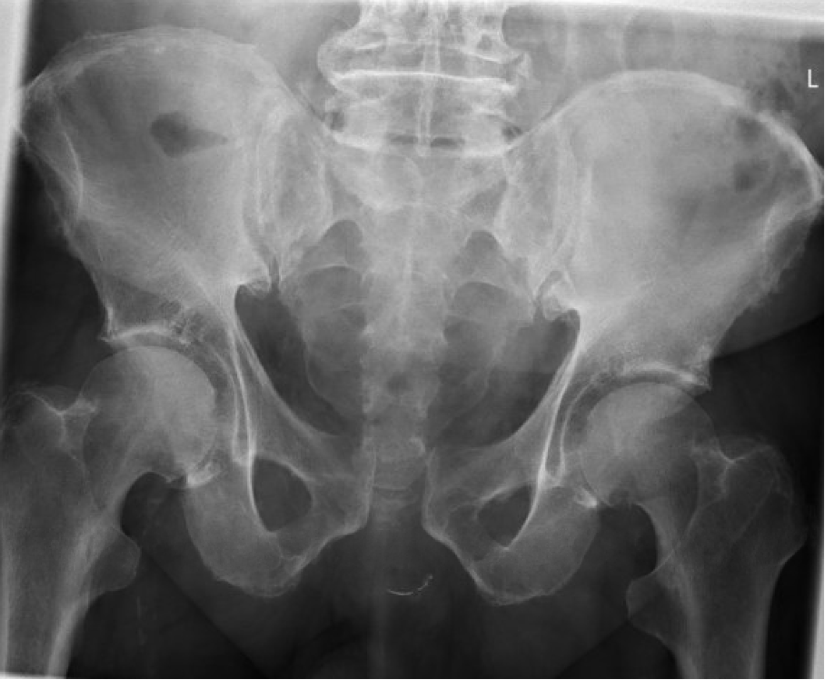 |
| Axial CT Scan:  * Double-click the CT image to view the video | 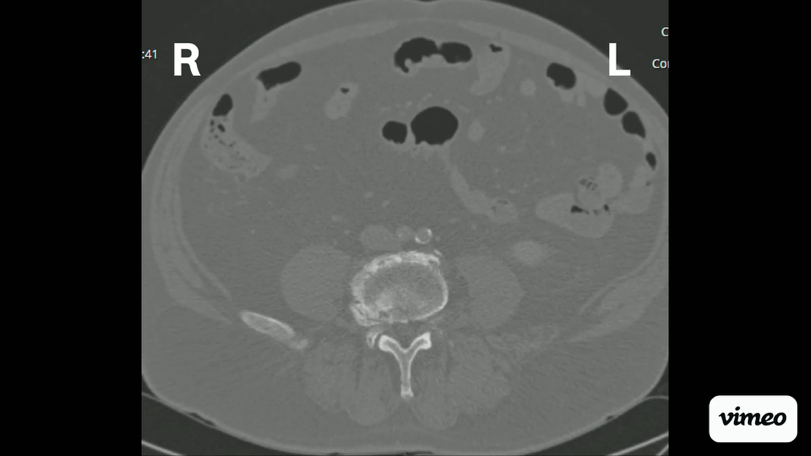 |
| Endorotation Stress (lateral compression): | 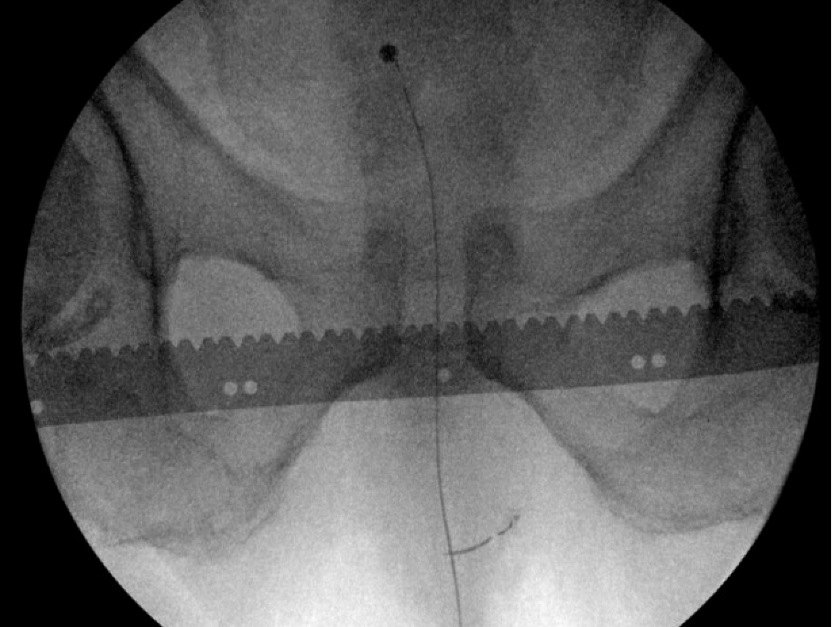 |
| Exorotation Stress (frog position): | 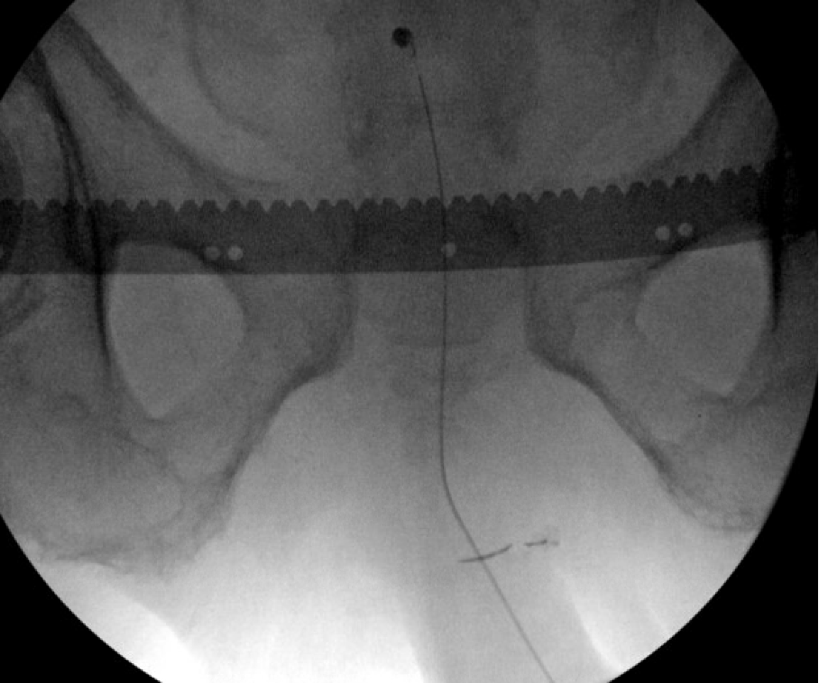 |
| Push right leg/ pull left leg: | 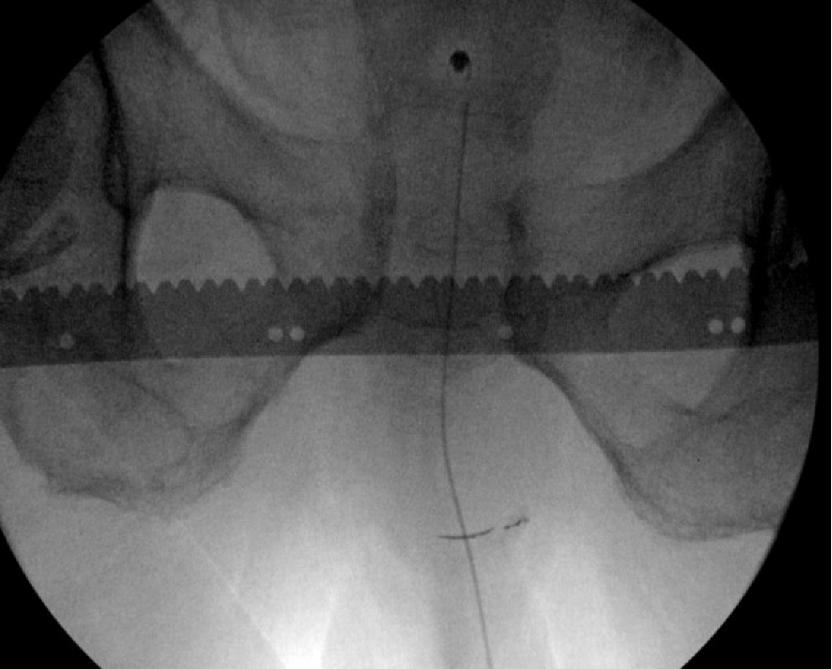 |
| Push left leg/ pull right leg: | 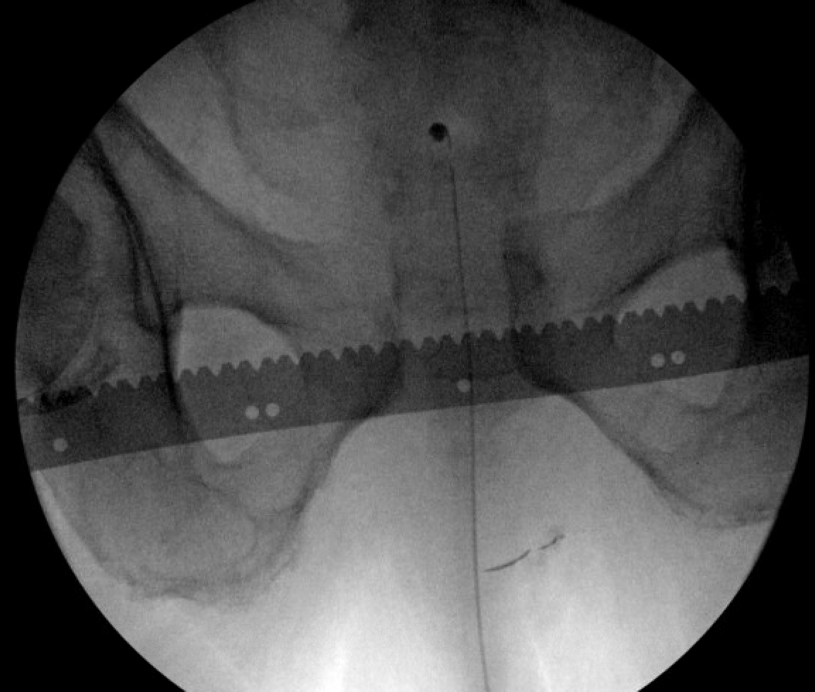 |
