# Supplementary figures and images for: Examination under anesthesia imaging changes surgeons’ classification and treatment decisions of anterior posterior compression pelvic ring injuries
Source: Eur J Orthop Surg Traumatol. 2026 May 30;36(1):202. doi: 10.1007/s00590-026-04744-8 (PMC13222183; doi:10.1007/s00590-026-04744-8)

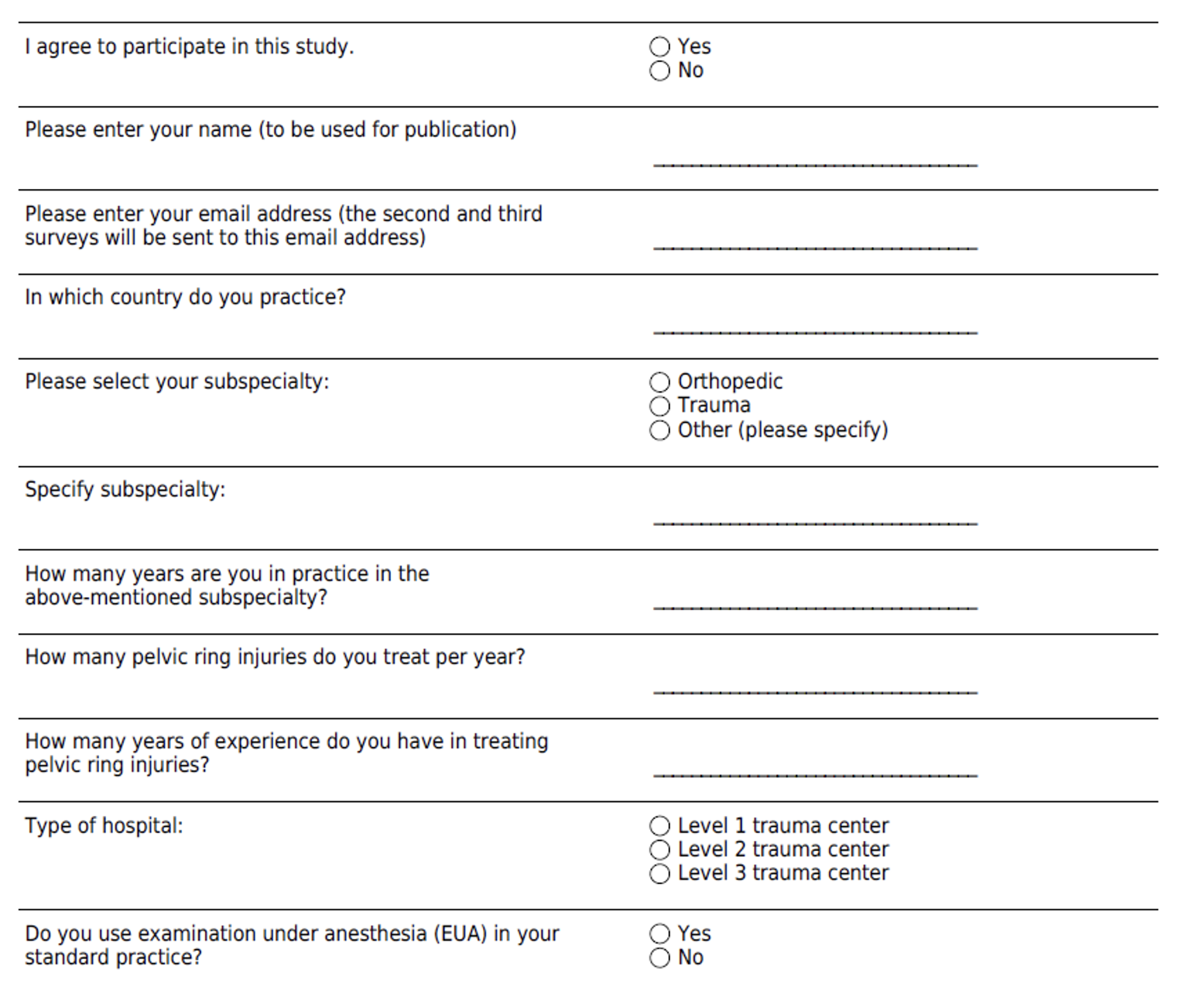

Supplement: Supplementary file 3 — Supplementary Material 3 [file 590_2026_4744_MOESM3_ESM.png]

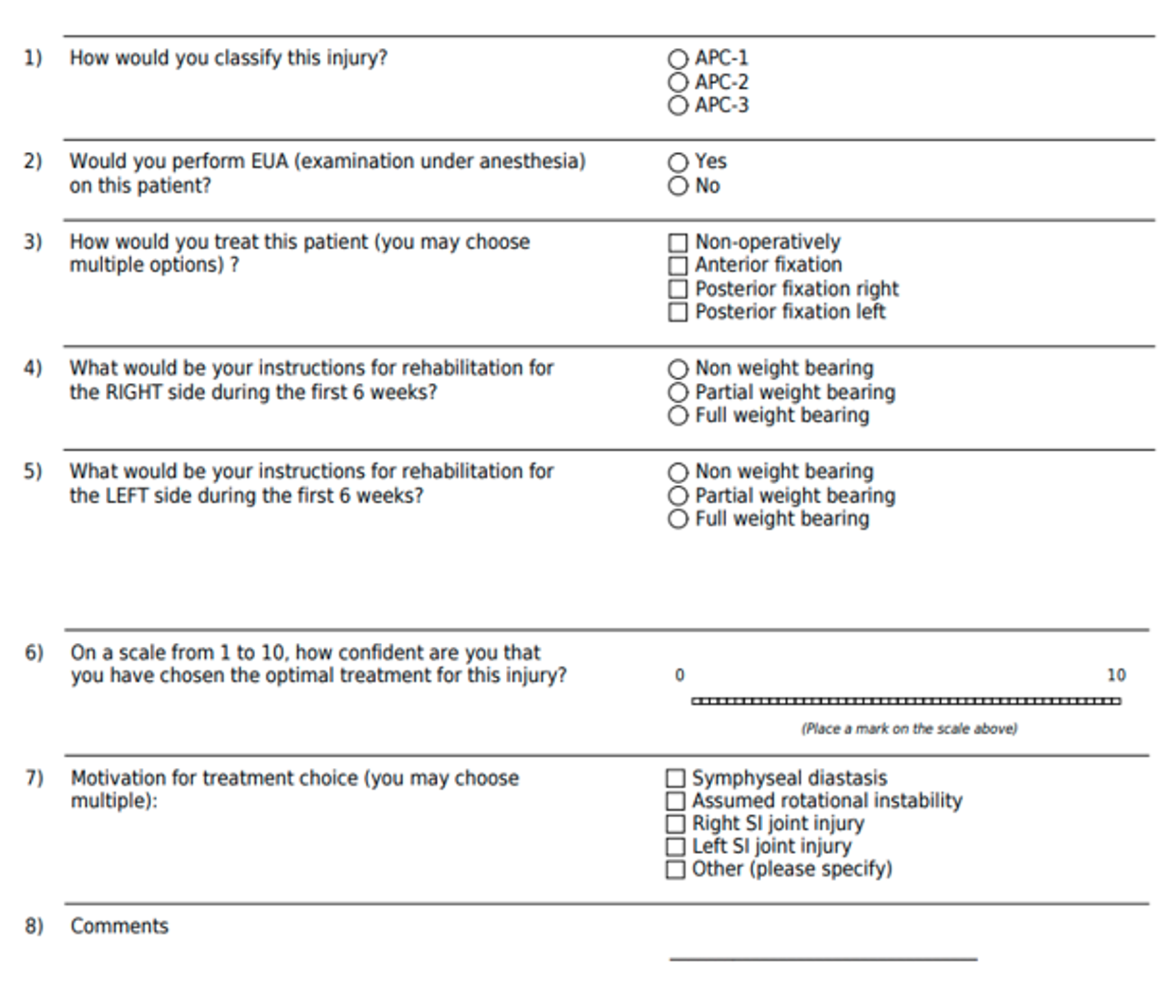

Supplement: Supplementary file 4 — Supplementary Material 4 [file 590_2026_4744_MOESM4_ESM.png]
