# Supplementary material for: Examination under anesthesia imaging changes surgeons’ classification and treatment decisions of anterior posterior compression pelvic ring injuries
Source: Eur J Orthop Surg Traumatol. 2026 May 30;36(1):202. doi: 10.1007/s00590-026-04744-8 (PMC13222183; doi:10.1007/s00590-026-04744-8)
Supplement: Supplementary file 5 — Supplementary Material 5 [file 590_2026_4744_MOESM5_ESM.docx]

**Appendix 5** Sub-analysis of surgeons who perform EUA in their standard practice vs those who do not.

| Case | 1 | 2 | 3 | 4 | 5 | 6 | 7 | 8 | 9 | 10 |
| --- | --- | --- | --- | --- | --- | --- | --- | --- | --- | --- |
| Change of classification EUA users, n (%) | 31 (58) | 19 (35) | 31 (58) | 15 (28) | 43 (78) | 20 (38) | 17 (32) | 22 (40) | 15 (27) | 23 (43) |
| Change of classification non- EUA users, n (%) | 39 (74) | 25 (47) | 32 (60) | 26 (48) | 39 (71) | 11 (21) | 22 (42) | 27 (51) | 16 (24) | 27 (51) |
| **P-value** | 0.422 | 0.127 | 0.500 | **0.019** | 0.369 | **0.043** | 0.210 | 0.171 | 0.451 | 0.280 |
| Change of treatment group EUA users, n (%) | 26 (50) | 7 (13) | 20 (42) | 17 (31) | 40 (75) | 24 (45) | 12 (23) | 14 (27) | 14 (26) | 21 (40) |
| Change of treatment group non-EUA users, n (%) | 19 (37) | 7 (14) | 14 (31) | 17 (33) | 44 (86) | 14 (28) | 10 (20) | 17 (35) | 18 (35) | 25 (47) |
| **P-value** | 0.117 | 0.535 | 0.200 | 0.530 | 0.125 | 0.053 | 0.445 | 0.264 | 0.223 | 0.278 |
