# Supplementary material for: Examination under anesthesia imaging changes surgeons’ classification and treatment decisions of anterior posterior compression pelvic ring injuries
Source: Eur J Orthop Surg Traumatol. 2026 May 30;36(1):202. doi: 10.1007/s00590-026-04744-8 (PMC13222183; doi:10.1007/s00590-026-04744-8)
Supplement: Supplementary file 6 — Supplementary Material 6 [file 590_2026_4744_MOESM6_ESM.docx]

**Appendix 6** Sub-analysis of surgeons who treat 50 or more pelvic injuries vs those who treat less than 50 per year.

| Case | 1 | 2 | 3 | 4 | 5 | 6 | 7 | 8 | 9 | 10 |
| --- | --- | --- | --- | --- | --- | --- | --- | --- | --- | --- |
| Change of classification ≥ 50 pelvic injuries per year, n (%) | 28 (49) | 20 (35) | 32 (56) | 14 (25) | 46 (79) | 14 (25) | 21 (37) | 24 (41) | 13 (22) | 24 (42) |
| Change of classification < 50 pelvic injuries per year, n (%) | 32 (57) | 24 (48) | 31 (63) | 27 (54) | 36 (72) | 17 (35) | 18 (37) | 25 (50) | 18 (36) | 26 (53) |
| **P-value** | 0.069 | 0.109 | 0.293 | **0.002** | 0.254 | 0.176 | 0.576 | 0.241 | 0.090 | 0.176 |
| Change of treatment group ≥ 50 pelvic injuries per year, n (%) | 21 (38) | 7 (12) | 20 (38) | 16 (28) | 45 (80) | 15 (26) | 13 (23) | 19 (35) | 14 (24) | 23 (40) |
| Change of treatment group < 50 pelvic injuries per year, n (%) | 24 (49) | 7 (15) | 14 (35) | 18 (37) | 39 (83) | 23 (50) | 9 (19) | 12 (26) | 18 (38) | 23 (47) |
| **P-value** | 0.181 | 0.440 | 0.480 | 0.228 | 0.396 | **0.012** | 0.417 | 0.242 | 0.101 | 0.314 |
